# Similar Properties of Chondrocytes from Osteoarthritis Joints and Mesenchymal Stem Cells from Healthy Donors for Tissue Engineering of Articular Cartilage

**DOI:** 10.1371/journal.pone.0062994

**Published:** 2013-05-09

**Authors:** Amilton M. Fernandes, Sarah R. Herlofsen, Tommy A. Karlsen, Axel M. Küchler, Yngvar Fløisand, Jan E. Brinchmann

**Affiliations:** 1 The Norwegian Center for Stem Cell Research, University of Oslo, Oslo, Norway; 2 Institute of Immunology, Oslo University Hospital Rikshospitalet, Oslo, Norway; 3 Department of Hematology, Oslo University Hospital Rikshospitalet, Oslo, Norway; University Hospital of Modena and Reggio Emilia, Italy

## Abstract

Lesions of hyaline cartilage do not heal spontaneously, and represent a therapeutic challenge. *In vitro* engineering of articular cartilage using cells and biomaterials may prove to be the best solution. Patients with osteoarthritis (OA) may require tissue engineered cartilage therapy. Chondrocytes obtained from OA joints are thought to be involved in the disease process, and thus to be of insufficient quality to be used for repair strategies. Bone marrow (BM) derived mesenchymal stem cells (MSCs) from healthy donors may represent an alternative cell source. We have isolated chondrocytes from OA joints, performed cell culture expansion and tissue engineering of cartilage using a disc-shaped alginate scaffold and chondrogenic differentiation medium. We performed real-time reverse transcriptase quantitative PCR and fluorescence immunohistochemistry to evaluate mRNA and protein expression for a range of molecules involved in chondrogenesis and OA pathogenesis. Results were compared with those obtained by using BM-MSCs in an identical tissue engineering strategy. Finally the two populations were compared using genome-wide mRNA arrays. At three weeks of chondrogenic differentiation we found high and similar levels of hyaline cartilage-specific type II collagen and fibrocartilage-specific type I collagen mRNA and protein in discs containing OA and BM-MSC derived chondrocytes. Aggrecan, the dominant proteoglycan in hyaline cartilage, was more abundantly distributed in the OA chondrocyte extracellular matrix. OA chondrocytes expressed higher mRNA levels also of other hyaline extracellular matrix components. Surprisingly BM-MSC derived chondrocytes expressed higher mRNA levels of OA markers such as *COL10A1*, *SSP1* (osteopontin), *ALPL*, *BMP2, VEGFA*, *PTGES, IHH*, and *WNT* genes, but lower levels of *MMP3* and *S100A4*. Based on the results presented here, OA chondrocytes may be suitable for tissue engineering of articular cartilage.

## Introduction

Articular cartilage defects have been reported in up to 20% of all patients undergoing arthroscopy of the knee [1]–[4]. Lesions of hyaline articular cartilage heal poorly. Untreated defects will usually give rise to pain, functional impairment, and eventually osteoarthritis (OA) [5], [6]. However, OA may also arise as a primary degenerative lesion of the hyaline cartilage [7]. Several treatment modalities have been developed over the past decades in attempts to reestablish fully functional cartilage tissue [8]. Cartilage cell therapy was introduced by using autologous chondrocyte implantation (ACI) [9]. However, the repair tissue generated following ACI frequently contains fibrocartilage, and ACI has not proven to be superior to other surgical techniques [10]–[12]. Unfortunately, OA patients frequently end up having total joint replacement surgery.

Tissue engineering combines the use of cells and biomaterials for repair of damaged tissues [13]. This approach has emerged as a promising strategy for cartilage repair [14]. In order to optimize this strategy, we need to determine which cell population to use, the best biomaterial, and whether to use growth- and differentiation factors or other differentiation promoting stimuli. The cells most likely to produce hyaline extracellular matrix (ECM) are articular chondrocytes and mesenchymal stem cells (MSCs) [15]–[17]. Chondrocytes are obtained from biopsies taken from the periphery of the articular cartilage in the course of arthroscopy. This creates a small lesion which also heals poorly, and may occasionally induce donor-site morbidity [8], [18]. However, the chondrocytes have already produced perfect hyaline ECM within the joint, and should in theory be able to do so again. In order to be useful for treatment of large cartilage defects, they need to be expanded *in vitro*. It is known that chondrocytes dedifferentiate when cultivated conventionally as monolayer cells [19]. This is the reason why ACI tends to produce fibrocartilage repair tissue. For chondrocytes obtained from OA joints, an additional problem arises. Here, all the chondrocytes within the joint are thought to be involved in the disease process [20]. Although all the molecular events involved in OA pathogenesis are not known, many of the markers describing chondrocyte hypertrophy during embryogenesis are also seen in OA chondrocytes [21]. During embryogenesis, chondrocyte hypertrophy is thought to be followed by apoptosis, vasculogenesis, and bone formation [22]. If any of these events should occur in chondrocytes used for tissue engineering of cartilage, this would affect the usefulness even of chondrocytes harvested away from the visible lesion in OA joints for engineering of hyaline cartilage for transplantation therapy.

MSCs are thought to resemble the progenitor cells responsible for the cartilage anlagen during embryological bone and cartilage formation [23]. Given the right differentiation clues they should be able to produce perfect hyaline ECM. MSCs may be obtained from BM with minimal discomfort and no residual morbidity, and are easily expanded *in vitro* [24]. Importantly, MSCs have been described as being immunoprivileged cells with immunosuppressive properties [25], which suggest that allogeneic BM-MSCs from young, healthy donors may represent an off-the-shelf choice of cells for tissue engineering of repair cartilage for OA patients. We have recently published a detailed description of gene and protein expression of human BM-MSCs in alginate hydrogel discs undergoing *in vitro* chondrogenic differentiation during exposure to differentiation medium [17]. Here we showed that mRNA encoding type II collagen (COL2) and other chondrogenic proteins, such as aggrecan (ACAN) and SOX5, 6 and 9, were quickly and highly expressed. However, mRNA coding for COL10 was also expressed. COL10 has been described in the literature as a marker for chondrocyte hypertrophy in limb development and endochondral bone formation [26]. Thus, both OA chondrocytes and BM-MSCs may be able to unleash genetic programs similar to those involved in embryonic chondrocyte hypertrophy.

Despite the obvious roles of chondrocytes and MSCs as the cell candidates for tissue engineering of hyaline cartilage for OA patients, to the best of our knowledge, no direct comparison has been made between human populations of these cells under identical differentiation conditions in biomaterial/cell cultures. Thus, in the present study we used our recently established culture system to expand OA chondrocytes *in vitro* [27]. These cells were subsequently differentiated in alginate discs under conditions that were identical to those used in our study of BM-MSCs which was performed at the same time [17]. Using material from the two cell populations in parallel analyses, we compared the kinetics of expression of hyaline cartilage-specific ECM genes between the two cell populations, and demonstrated synthesis of ECM proteins and glycosaminoglycans. Finally, we performed a microarray comparison of the global gene expression between OA chondrocytes and BM-MSCs differentiated in alginate hydrogel discs under identical conditions. The results show that the OA chondrocytes produced more hyaline ECM components, expressed lower levels of many of the markers for chondrocyte hypertrophy, bone differentiation, and vasculogenesis. Thus, OA chondrocytes may be suitable for tissue engineering of articular cartilage.

## Materials and Methods

### Chemicals

All the chemicals were purchased from Sigma-Aldrich (St. Louis, MO) unless otherwise stated.

### Isolation and Culture of the Chondrocytes

Biopsies of articular cartilage were provided from OA patients with primary OA undergoing knee replacement surgery. All the cartilage biopsies were taken from a part of the surface of the femoral condyle considered by the surgeon to look like intact and healthy cartilage. The three donors were 60–65 years of age, and all provided written informed consent. The study was approved by the Regional Committee for Medical Research Ethics, Southern Norway, Section A. The biopsies were cut into tiny pieces and digested with Collagenase type XI (1.2 mg/mL) at 37°C for 90–120 minutes. The digested cartilage pieces were then washed and resuspended in Dulbecco’s modified Eagle’s medium (DMEM)-F12 (Gibco, Paisley, UK) supplemented with 20% fetal bovine serum (FBS, Cambrex, East Rutherford, NJ), 50 µg/mL ascorbic acid, 1% penicillin and streptomycin (P/S), and 1.5 µg/mL amphotericin B. The culture medium was changed every 3–4 days. At 70–80% confluence, cells were detached with trypsin/EDTA and seeded into new culture flasks. 25 cm^2^ culture flasks (Nunc, Roskilde, Denmark) were used for the first passage, before proceeding with 175 cm^2^ flasks. Amphotericin B was discontinued and FBS was reduced to 10% after the first passage.

### Isolation and Culture of Mesenchymal Stem Cells from Bone Marrow

The mononuclear cell fractions were isolated from human bone marrows of three healthy, voluntary donors aged 24–50 years by density gradient centrifugation (Lymphoprep, Fresenius Kabi, Oslo, Norway) as described [17]. The cells were seeded in 175-cm^2^ tissue culturing flasks and cultured in expansion medium containing DMEM-F12, 1% PS, 2.5 µg/ml amphotericin B, and 10% fetal bovine serum. After 48 hours the non-adherent cells were removed by medium exchange. The adhering cells were expanded in monolayer culture with medium change twice a week until colonies reached 70% confluence. Cultures were passaged using Trypsin/EDTA and reseeded at 5000 cells/cm^2^. After the first passage amphotericin B was removed from the culture medium. For practical purposes the cells were frozen at passage 2 in DMEM-F12 containing 20% FBS and 5% DMSO. After thawing, cells were reseeded at a density of 5000 cells/cm^2^ and passaged when 70% confluent.

### Chondrogenic Differentiation in 3D Alginate Scaffold

At passage 2–3, after 18–20 days of cultivation for the OA chondrocytes, and at passage 3–4 for the BM-MSCs, the cells were embedded in equal amounts of 1% Pronova LVG-alginate and 1% calcium alginate in a 4.6% mannitol solution. This scaffold is a self-gelling system provided by FMC BioPolymer AS/NovaMatrix (Sandvika, Norway) [17], [28], [29]. The alginate gels were shaped into 0.8 mL discs containing 5×10^6^ cells/mL in 12 well culture plates (Nonstick, Nunc). After washing with DMEM, the discs were washed with a 50 mM SrCl_2_ solution while solidifying. After solidification the scaffolds were again washed with DMEM medium and moved to 6 well culture plates (Nonstick, Nunc). After establishment of the cells in the alginate discs, chondrogenic differentiation was induced by high-glucose DMEM (4.5 g/l) supplemented with 1 mM sodium pyruvate (Gibco), 0.1 mM ascorbic acid-2-phosphate, 0.1 µM dexamethasone, 1% ITS (insulin 25 µg/ml, transferrin 25 µg/ml, and sodium selenite 25 ng/ml), 1.25 mg/ml human serum albumin (Octapharma, Hurdal, Norway), 500 ng/ml bone morphogenic protein-2 (BMP-2, InductOs, Wyeth, Taplow, UK), and 10 ng/ml recombinant human transforming growth factor-ß1 (TGF-β1, R&D Systems, Minneapolis, MN). Medium was changed every day for the first three days, then every 2–4 days. During expansion and differentiation cultures the same batches of all supplements, FBS, differentiation reagents, and alginate were used for the two cell populations.

### Total RNA Isolation

The alginate scaffolds were depolymerized on a thermo shaker 37°C for 30 minutes using 5 U/mL of the enzyme GLyase (kindly donated by Professor Gudmund Skjåk-Bræk). 1 mL GLyase solution was used to treat 400 µL of the alginate discs. RNA was isolated from the cells using the TRIzol method following the company’s protocol (Invitrogen, Carsbad, CA). The cell samples were dissolved in TRIzol, frozen in liquid nitrogen, and stored at −80°C.

### Real-time Reverse Transcription-polymerase Chain Reaction Quantification

Isolated total RNA was treated with DNase I (Ambion, Austin, TX) followed by quantification with NanoDrop ND-1000 Spectrophotometer (Nanodrop Technologies, Wilmington, DE). 200 ng of total RNA from each sample was reverse transcribed into cDNA (total volume of 20 µL) using High Capacity cDNA Reverse Transcription kit (Applied Biosystems, Abingdon, UK) following the manufacturer’s protocol. The cDNA samples were analyzed by relative quantification using the 7300 Real-Time reverse transcriptase quantitative PCR (RT-qPCR) System (Applied Biosystems) and TaqMan® Gene Expression assay following manufacture’s protocol. Primers from Applied Biosystems are listed in Table 1. *B2M* proved to be the most stably expressed gene within the differentiated OA chondrocytes and BM-MSCs, and was therefore used as the endogenous control. All the expression levels were normalized to the expression of the endogenous control.

**Table 1:** Taqman assay primers used in real-time reverse transcription polymerase chain reaction and antibodies used in immunohistochemistry.

		*Primers*	*Antibodies*		
*Gene symbol*	*Protein*	*Taqman assay no.*	*Designation (concentration)*	*Type and Ig class*	*Source*
*ACAN*	Aggrecan	Hs00202971_m1	969D4D11 (2.28 µg/mL)	Mouse monoclonal IgG1	BioSource
*ALPL*	Alkaline phosphatase	Hs00758162_m1	–	–	–
*B2M*	Beta-2 microglobulin	Hs99999907_m1	–	–	–
*COL1A1*	Collagen type I	Hs00164004_m1	I-8H5 (1.00 µg/mL)	Mouse monoclonal IgG2a	MP Biomedicals
*COL2A1*	Collagen type II	Hs00156568_m1	II-4C11 (0.83 µg/mL)	Mouse monoclonal IgG1	MP Biomedicals
*COL3A1*	Collagen type III	Hs00943809_m1	–	–	–
*COL9A1*	Collagen type IX	Hs00156680_m1	–	–	–
*COL10A1*	Collagen type X	Hs00166657_m1	X53 (1∶200)	Mouse monoclonal IgG1	Prof. Klaus von der Mark
*COMP*	Cartilage oligomeric matrix protein	Hs00164359_m1	–	–	–
*FN1*	Fibronectin 1	Hs01549976_m1	–	–	–
*IBSP*	Integrin-binding sialoprotein/bone sialoprotein	Hs00173720_m1	–	–	–
*MMP13*	Matrix metalloproteinase 13	Hs00233992_m1	–	–	–
*MMP3*	Matrix metalloproteinase 3	Hs00867308_m1	–	–	–
*RUNX2*	Runt-related transcription factor 2	Hs00231692_m1	–	–	–
*SOX5*	SRY (sex determining region Y)-box containing gene 5	Hs00374709_m1	ab26041 (1.42 µg/mL)	Rabbit polyclonal	Abcam
*SOX6*	SRY (sex determining region Y)-box containing gene 6	Hs00264525_m1	HPA001923 (1.67 µg/mL)	Rabbit polyclonal	Sigma
*SOX9*	SRY (sex determining region Y)-box containing gene 9	Hs00165814_m1	AB5535 (0.20 µg/mL)	Rabbit polyclonal	Millipore
*SSP1*	Secreted phosphoprotein 1/bone sialoprotein 1/osteopontin	Hs00959010_m1	–	–	–
*VCAN*	Versican	Hs01007941_m1	255915 (2.50 µg/mL)	Rat monoclonal IgG1	R&D

### Fluorescence Immunohistochemistry

All antibodies and concentrations are listed in Table 1. The following secondary antibodies were used: Alexa 488-conjugated goat anti-rabbit antibody (used at 5 µg/mL), purchased from Invitrogen, and Cy3-conjugated donkey anti-rat IgG (used at 2 µg/mL) and Cy3-conjugated donkey anti-mouse IgG (used at 1.4 µg/mL), both purchased from Jackson Immuno Research Europe (Newmarket, UK). Formalin-fixed, paraffin-embedded 3D culture samples from day 21 were sectioned and deparaffinised, using standard laboratory procedures, and postfixed for 10 minutes in 4% paraformaldehyde in phosphate-buffered saline (PBS; Electron Microscopy Sciences, Hatfield, PA). Tissue sections were boiled for 12 minutes in antigen retrieval buffer. TrisEDTA buffer (pH 9.0) was used for antibody ab26041, staining for SOX5, and 0.05% citraconic anhydride in ddH2O (pH 7.4) was used for all the other antibodies. One section per slide was incubated with primary antibodies diluted in 1.25% bovine serum albumin with 0.1% saponin in PBS. The other section served as negative control and was incubated with the same buffer without antibody. Sections were incubated overnight at 4°C. Subsequently, secondary fluorochrome-conjugated antibodies were applied to both sections for 1.5 hours at room temperature. Sections were mounted with ProLong Gold antifading reagent with DAPI (Invitrogen), two sections per slide, one with and one without primary antibody. Microscopy was performed with a Nikon Eclipse E-600 fluorescence microscope equipped with Nikon Plan-Fluor objective lenses and Color View III digital camera controlled by Cell-B software (Olympus; www.olympusglobal.com/en/). No fluorescence signal was detectable in negative control sections. Analysis of signal, specific for the primary antibodies used, was performed with automatic camera settings, to enable optimal visualization of structures.

### Microarray Analysis

RNA samples from chondrogenically differentiated OA chondrocytes and BM-MSCs at day 21 were analyzed at the Norwegian Microarray Consortium according to the manufacturer’s protocol. Biotin labeled cRNA was transcribed using Illumina® TotalPrep RNA Amplification Kit (Ambion). cRNA hybridized onto Illumina HumanWG-6 v3 Expression BeadChips was subsequently stained with streptavidin-Cy3. The chips were then scanned using Illumina® BeadArray™ Reader. Results were imported and quantile normalized in Illumina GenomeStudio v. 2009.1 Gene Expression v. 1.1.1. for data extraction and initial quality control by using the array annotation file “HumanWG-6_V3_0_R3_11282955_A.bgx”. Further quality control, reprocessing, log(2) transformation and expression analysis were performed in the microarray analysis program J-Express 2009 [30]. The quality control consisted of box plot, correspondence analysis plot, and hierarchical clustering with distance matrix. The statistical analysis was performed using Rank Product (RP) [31] for calculating the differential expression between the two groups. A cut-off value was set at >2 fold expression change. RP’s own q-value (adjusted p-value) <0.02 for both positive and negative scores was used to produce two lists representing the upregulation-values for the two cell groups. Gene Ontology [32] (GO) overrepresentation analysis, using Bonferroni correction (p<0.05), was then performed on the genes that were found differently expressed by RP.

### Statistical Analysis

Differences in gene expression measured by RT-qPCR in OA chondrocytes and BM-MSCs were evaluated using unpaired Student’s t-test, assuming Gaussian distribution. p<0.05 was considered significant.

## Results

### Gene Expression Analysis

**Figure 1:**
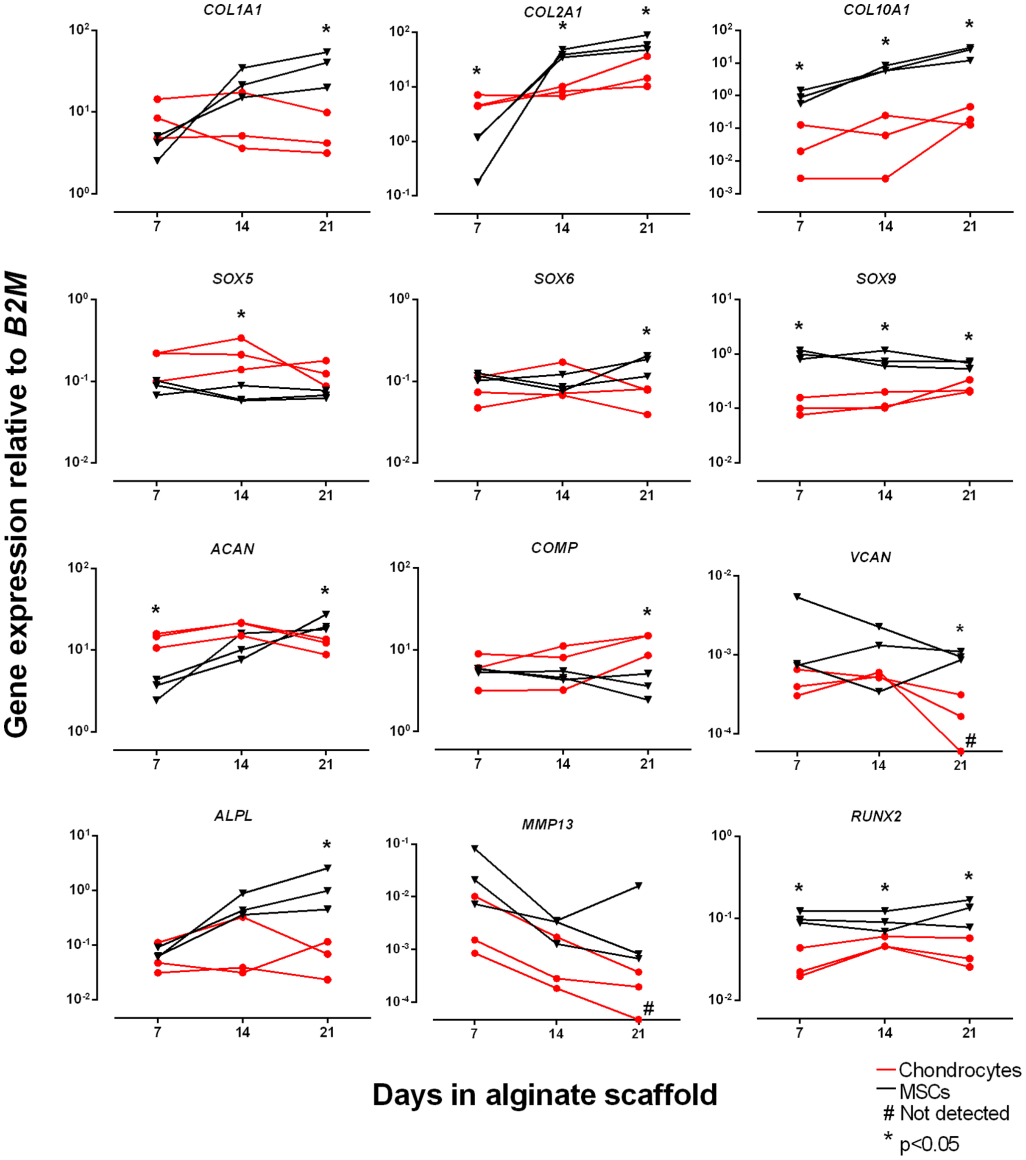
Real-time reverse transcription-polymerase chain reaction quantification (RT-qPCR) of genes expressed in human osteoarthritis (OA) chondrocytes and human bone-marrow (BM) mesenchymal stem cells (MSCs) embedded in self-gelling alginate scaffold with chondrogenic medium. Quantification was made on days 7, 14, and 21. The gene expression was normalized to *Beta-2 microglobulin* (*B2M*) expression levels. The mRNA gene expression levels for the OA chondrocytes are represented by the red lines and circular symbols, and the BM-MSC derived chondrocytes by the black lines and triangular symbols. Each data point represents the mean of technical triplicates, and each line represents one of three donors run in parallel. “#” signifies non-detectable levels of expression. * signifies statistically significant difference between cell populations (p<0.05).

RT-qPCR analysis for a number of selected genes expressed in chondrogenically differentiated OA chondrocytes and BM-MSCs, which had been cultivated under identical conditions in alginate scaffolds, are presented in Figure 1. For the collagen genes, *COL1A1* remained quite stable, while the expression of *COL2A1* and *COL10A1* increased slightly from day 7 to day 21 in the OA chondrocytes. The expression of these genes in the differentiating BM-MSCs, however, increased considerably in this period. The difference between the BM-MSCs and the OA chondrocytes for mean expression of *COL1A1*, *COL2A1*, and *COL10A1* at day 21 were approximately 6 fold, 3 fold and 85 fold, respectively. The expression of *COL2A1* on day 7 was considerably higher in OA chondrocytes than in BM-MSC derived chondrocytes, consistent with retained expression of *COL2A1* in the chondrocytes in the course of *in vitro* culture.

The SOX transcription factors *SOX5* and *SOX6* mRNA had similar expression levels when comparing the OA and BM-MSC derived chondrocytes, and with relatively little change in expression over the measured time points. *SOX9*, however, was consistently expressed at higher levels in the BM-MSC derived chondrocytes compared to OA chondrocytes. The expression of the non-collagenous matrix protein genes *ACAN*, *COMP*, and *VCAN* were grossly unaltered over time in the chondrocytes. In the BM-MSC derived chondrocytes, however, *ACAN* expression showed approximately 5-fold increase between days 7 and day 21, starting at much lower levels than in the OA chondrocytes. The gene markers of hypertrophic chondrocytes *ALPL*, *MMP13*, and *RUNX2* were also investigated and compared between the cell types. The differentiating BM-MSCs had a 10-fold increase in *ALPL* gene expression between days 7 and 21, with a considerably higher expression level in the BM-MSC derived chondrocytes than in the OA chondrocytes at day 21. *MMP13* had a rather similar trend in both OA and BM-MSC derived chondrocytes with a 10-fold decrease in expression levels between day 7 and 21, with a trend towards lower expression in the OA chondrocytes. The expression level of MMP13 at the end of the culture period was very low. *RUNX2* showed a higher level of expression in the BM-MSC derived chondrocytes over the measured time points, though both cell types showed a rather stable expression profile.

### Protein Expression Analysis

**Figure 2:**
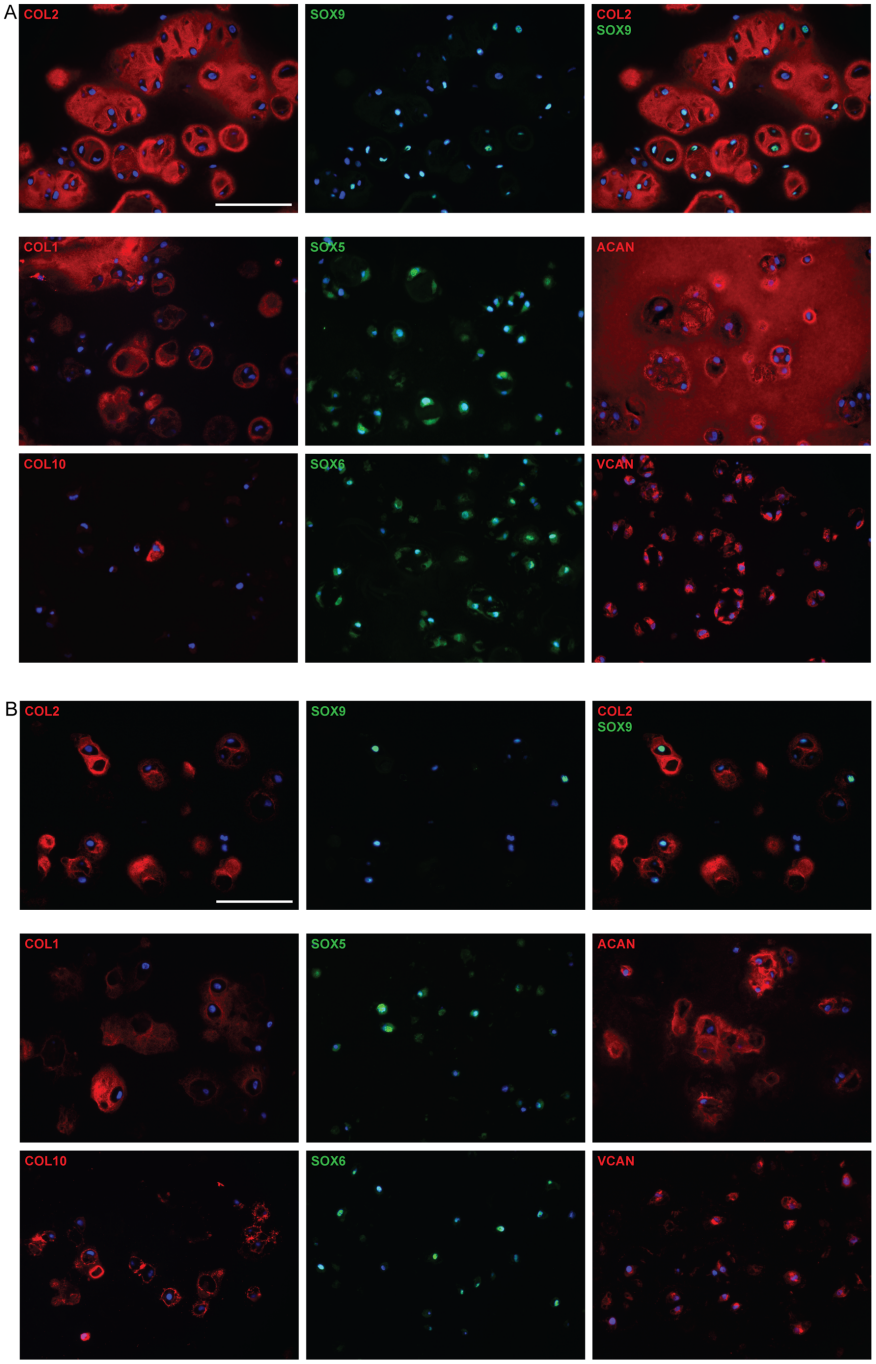
Fluorescence immunohistochemical analysis of protein expression in OA chondrocytes (A) and BM-MSC derived chondrocytes (B) embedded in alginate scaffold with chondrogenic medium for 21 days. COL2 (red color) and SOX9 (green color) were co-stained and is shown both as separate images and in overlay. COL1, COL10, ACAN and VCAN are shown in red color, whereas SOX5, SOX6, and SOX9 are shown in green color. Cellular nuclei were counterstained with DAPI (blue). Scale bar = 100 µm, applies to all images.

Fluorescence immunohistochemistry images of the chondrogenically differentiated OA chondrocytes and BM-MSCs embedded in alginate scaffold on day 21 are shown in Figure 2A and B, respectively. COL2 was found to surround nearly all the cells of both cell types. SOX9 was detected as a nuclear signal in more than half of the cells. We found no correlation between the nuclear SOX9 expression signal and the amount of extracellular COL2 signal in either of the cell types. We found both SOX9 positive and negative cells with and without extracellular COL2 signal. COL1 was detected as an extracellular signal around more than half of the cells in both cell populations. Interestingly, a major difference was observed between the two cell populations for the synthesis of COL10. Among the OA chondrocytes extremely few cells were positive for COL10, which stained only in the cytoplasm. For the BM-MSC derived chondrocytes, however, COL10 was found to be synthesized by the vast majority of the cells, and was stained both in the cytoplasm and in the immediate pericellular region at this time point. The expression of the transcription factor proteins SOX5 and SOX6 was similar for the two cell populations, and appeared to be higher than the fraction of SOX9 positive cells. ACAN was found to be synthesized and secreted by almost all the cells within both populations, with a trend towards a more generalized intercellular distribution in the OA chondrocytes. Surprisingly, VCAN demonstrated a strong cytoplasmic distribution in many cells, despite low expression on the RT-qPCR. Little VCAN was detected in the ECM.

### Comparison of mRNA Microarray Analysis

Evaluation of differential gene expression by RP analysis showed that 427 genes were upregulated in the OA chondrocytes and 388 genes in the BM-MSC derived chondrocytes after 21 days of differentiation (Table S1 and S2). The GO distribution hierarchy trees for these genes are shown in Table S3. Following Bonferroni correction, the Extracellular Region category (GO: 0005576) came out as most significantly overrepresented GO term among the upregulated genes in the OA chondrocytes. This category was also significantly overrepresented in the BM-MSC derived chondrocytes. As this category was likely to include genes involved in the synthesis and regulation of the ECM, we chose to identify the 82 genes upregulated in OA chondrocytes and the 64 genes upregulated in BM-MSC derived chondrocytes in this category. These genes, subcategorized by ourselves, are presented in Table 2. Most surprisingly, a number of genes associated with hypertrophic chondrocytes and OA were found to be expressed at higher levels in differentiated BM-MSCs than in OA chondrocytes: *COL10A1*, *SSP1*, *ALPL*, *BMP2, VEGFA, IHH* and several *WNT* genes [21], [33]–[36]. The differences for the OA markers *MMP13* and *RUNX2*, observed using the more sensitive RT-qPCR assay, could not be verified here. Similarly, the differences observed for *COL2A1* and *COL1A1* by RT-qPCR could not be verified by the microarray method. Among genes associated with hyaline cartilage, *COL9A1*, *CLIP*, and *DCN* were expressed at higher levels in the OA chondrocytes, while *FN1* and *FBLN1* were expressed at higher levels in the BM-MSC derived chondrocytes. Genes encoding matrix metallopeptidases and ADAM metallopeptidases were upregulated in the OA chondrocytes, while a number of WNT pathway genes were upregulated in the differentiated BM-MSCs. In general, more genes encoding matrix molecules, and particularly hyaline matrix molecules were found to be upregulated in the OA chondrocytes on day 21 of chondrogenic differentiation in alginate. However, a number of genes encoding molecules important for hyaline cartilage, such as COL11, matrillin 3, biglycan, fibromodulin, hyaluronan, and proteoglycan link protein 1, were expressed at variable and similar levels in the two cell populations (data not shown).

**Table 2:** List of genes within the GO term Extracellular Region which were differentially expressed in OA chondrocytes and chondrogenically differentiated human BM-MSCs.

Upregulated in OA chondrocytes			Upregulated in differentiated BM-MSCs		
Symbol	Name	Fold increase	Symbol	Name	Fold increase
**Extracellular** **matrix proteins**			**Extracellular matrix proteins**		
COL3A1	Collagen type 3	5.7	COL27A1	Collagen type 27	2.6
COL9A1	Collagen type 9	2.8	COL10A1	Collagen type 10	6.6
COL16A1	Collagen type 16	2.5			
COL24A1	Collagen 24	2.5			
CLIP	Cartilage intermediate layer protein	2.9	IBSP	Integrin-binding sialoprotein/bone sialoprotein	9.6
CILP2	Cartilage intermediate layer protein 2	5.1	SPP1	Secreted phosphoprotein 1/bone sialoprotein 1/osteopontin	10.2
CRTAC1	Cartilage acidic protein 1	8.6	FN1	Fibronectin 1	2.6
MFAP4	Microfibrillar-associated protein 4	6.0	MATN4	Matrilin 4	8.7
CHI3L2	Chitinase 3-like 2	2.5			
FBLN5	Fibulin 5	4.3			
AMTN	Amelotin	6.4			
ELN	Elastin	4.3			
DPT	Dermatopontin	12.5			
MGP	Matrix Gla protein	3.8			
EFEMP1	EGF-containing fibulin-likeECM protein 1	4.1			
FNDC1	Fibronectin type III domaincontaining 1	3.6			
SMOC1	SPARC related modular calciumbinding 1	4.3	SPARCL1	SPARC-like 1/hevin	3.3
SMOC2	SPARC related modular calciumbinding 2	2.8			
ECM2	Extracellular matrix protein 2	4.0			
LAMA2	Laminin alpha 2	2.3	LAMA4	Laminin alpha 4	4.2
**Glycoproteins,** **proteoglycans,** **and PG modifiers**			**Glycoproteins, proteoglycans, and PG modifiers**		
OGN	Osteoglycin	9.8	FBLN1	Fibulin	3.0
MAMDC2	MAM domain containing2/mamcan	5.5	GPC3	Glypican 3	5.8
SULF1	Sulfatase 1	2.6	GPC1	Glypican 1	3.3
OMD	Osteomodulin	4.9	ENG	Endoglin/CD105	3.0
DCN	Decorin	3.4	FSTL3	Follistatin-like 3	2.6
**Matrix remodeling**			**Matrix remodeling**		
ADAMTS1	ADAM metallopeptidase withthrombospondin 1 motif	2.3	A2M	Alpha 2 macroglobulin	5.6
ADAMTSL3	ADAMTS-like 3	5.4	LOX	Lysyl oxidase	3.8
MXRA5	Matrix-remodeling associated 5	4.3	LOXL4	Lysyl oxidase-like 4	3.3
SERPINA5	Serpin peptidase inhibitor,clade A, 5	5.2	LOXL3	Lysyl oxidase-like 3	2.6
SERPINA1	Serpin peptidase inhibitor,clade A, 1	7.0	SERPINE2	Serpin peptidase inhibitor, clade E, 2	5.6
SPINT2	Serine peptidase inhibitor,Kunitz type, 2	6.3	KAZALD1	Kazal-type serine peptidase inhibitor domain 1	2.8
SLPI	Secretory leukocyte peptidaseinhibitor	2.8	P4HB	Prolyl 4-hydroxylase, beta	2.6
MMP7	Matrix metallopeptidase 7	25.0	ALPL	Alkaline phosphatase	6.1
MMP3	Matrix metallopeptidase 3	21.1	MMP2	Matrix metallopeptidase 2	2.5
MMP23A	Matrix metallopeptidase 23A	2.8	PAMR1	Peptidase associated with muscle regeneration 1	14.1
			CTSD	Cathepsin D	8.4
**Growth factors, GF receptors, and GF antagonists**			**Growth factors and GF receptors**		
TNFRSF11B	Tumor necrosis factor receptor 11B/osteoprotegerin	11.0	IGFBP4	Insulin-like growth factor binding protein 4	4.0
IGFBP6	Insulin-like growth factor binding protein 6	6.4	IGFBP5	Insulin-like growth factor binding protein 5	14.6
GDF10	Growth differentiation factor 10	13.1	GDF15	Growth differentiation factor 15	6.6
PDGFD	Platelet derived growth factor D	3.3	VEGFA	Vascular endothelial growth factor A	4.4
GREM1	Gremlin 1	4.2	TGFBI	Transforming growth factor, beta-induced	5.1
TGFBR3	Transforming growth factor beta receptor 3	2.8	MDK	Midikine	2.5
BMP4	Bone morphogenetic protein 4	2.7	BMP2	Bone morphogenetic protein 2	3.6
			PGF	Placental growth factor	5.6
**Signaling pathways**			**Signaling pathways**		
CD47	CD47 molecule	2.4	WNT4	Wingless-type MMTV integration site family 4	4.9
WISP2	WNT1 inducible signaling protein 2	4.6	WNT5A	Wingless-type MMTV integration site family 5A	2.4
SFRP5	Secreted frizzled-related protein 5	4.0	WNT5B	Wingless-type MMTV integration site family 5B	4.5
JAG1	Jagged 1	2.6	WNT 11	Wingless-type MMTV integration site family 11	3.9
SCUBE2	Signal peptide, CUB domain, EGF-like 2	2.6	SFRP1	Secreted frizzled-related protein 1	3.1
			DKK1	Dickkopf 1	3.7
			IHH	Indian hedgehog	4.2
			PTGDS	Prostaglandin D2 synthase	3.4
			SCUBE3	Signal peptide, CUB domain, EGF-like 3	3.9
**Cytokines**			**Cytokines**		
CYTL1	Cytokine-like 1	36.8	MIF	Macrophage migration inhibitory factor	2.8
IL17RB	Interleukin 17 receptor B	2.4	CXCL14	Chemokine (C-X-C motif) ligand 14	14.0
CCL2	Chemokine (C-C motif) ligand 2	3.3			
**Cell adhesion**			**Cell adhesion**		
MSLN	Mesothelin	5.1	FLRT3	Fibronectin leucine rich transmembrane protein 3	2.8
SVEP1	Sushi, von Willebrand factor type A, EGF and pentraxin domain containing 1	7.6	CLEC3A	C-type lectin domain family 3 A	3.7
TNC	Tenascin C	7.7	CLEC11A	C-type lectin domain family 11 A	2.5
CHAD	Chondroadherin	3.1	LGALS3BP	Lectin, galactoside-binding, soluble, 3 binding protein	4.0
CDH13	Cadherin 13	2.3	THBS2	Thrombospondin 2	3.7
THBS3	Thrombospondin 3	4.6			
**Miscellaneous**			**Miscellaneous**		
CFH	Complement factor H	3.3	CFD	Complement factor D	3.8
CFB	Complement factor B	2.4	PZP	Pregnancy-zone protein	2.8
C5	Complement factor 5	3.6	PCSK5	Proprotein convertase subtilisin/kexin 5	2.5
ABI3BP	ABI gene family, member 3 binding protein	13.3	ALDOA	Aldolase A	2.7
RPESP	RPE-spondin	4.0	F12	Coagulation factor XII	2.7
WFDC1	WAP four-disulfide core domain 1	2.7	TUBA4A	Tubulin alpha 4A	2.7
DMKN	Dermokine	2.9	DHRS11	Dehydrogenase/reductase (SDR family) 11	3.0
TMSB4X	Thymosin, beta 4, X-linked	2.9	CCDC80	Coiled-coil domain containing 80	2.4
CA2	Carbonic anhydrase 2	3.7	SLIT3	Slit homolog 3	2.9
GPX3	Glutathione peroxidase 3	8.2	TF	Transferrin	3.6
ISM1	Isthmin 1 homolog	2.7	ACTN4	Actinin, alpha 4	2.6
PLA2G2A	Phospholipase A2, group 2A	8.3	ADM	Adrenomodulin	3.0
PON3	Paraoxonase 3	3.3	GPI	Glucose phosphate isomerase	3.1
POSTN	Periostin	4.6	HLA-C	Human leukocyte antigen class I, C	2.4
CPXM2	Carboxypeptidase X (M14 family) 2	2.3	MICA	MHC class I polypeptide-related sequence A	2.9
C2orf40	Chromosome 2 open reading frame 40	12.0			
OLFM1	Olfactomedin	5.4			
FST	Follistatin	2.5			
PRRG4	Proline rich Gla 4	2.6			
CNPY4	Canopy 4	2.3			
RAMP1	Receptor activity modifying protein 1	2.5			
ANGPT1	Angiopoietin 1	3.2			
ANGPTL7	Angiopoietin-like 7	4.7			
ANGPTL5	Angiopoietin-like 5	13.7			

**Figure 3:**
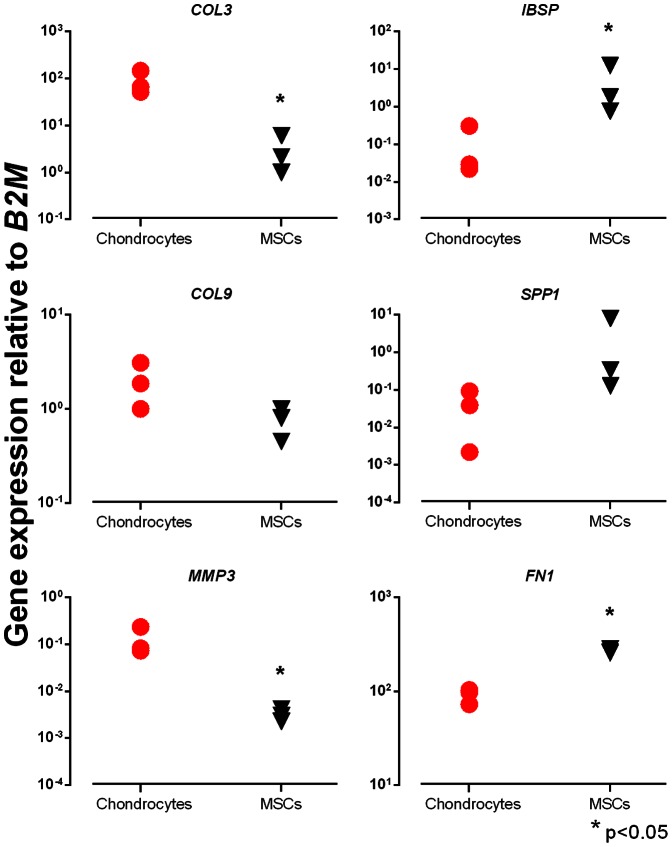
RT-qPCR verification of selected genes from the microarray comparison of the OA chondrocytes (red circles) and BM-MSC derived chondrocytes (black triangles) following 21 days of chondrogenic differentiation in alginate discs. Gene expression was normalized to *B2M* expression levels. Shown are mean values of the technical triplicates for each donor. * signifies statistically significant difference between cell populations (p<0.05).

We went on to verify the upregulation of some of the genes by RT-qPCR. *B2M* was again used as endogenous control. We analyzed the expression of *COL3A1*, *COL9A1*, *MMP3*, *IBSP*, *SSP1*, and *FN1*, and found that the changes observed in the microarray analysis could in all cases be confirmed, and that in most cases the fold change differences were greater in the RT-qPCR analysis. The results are shown in Figure 3.

**Figure 4:**
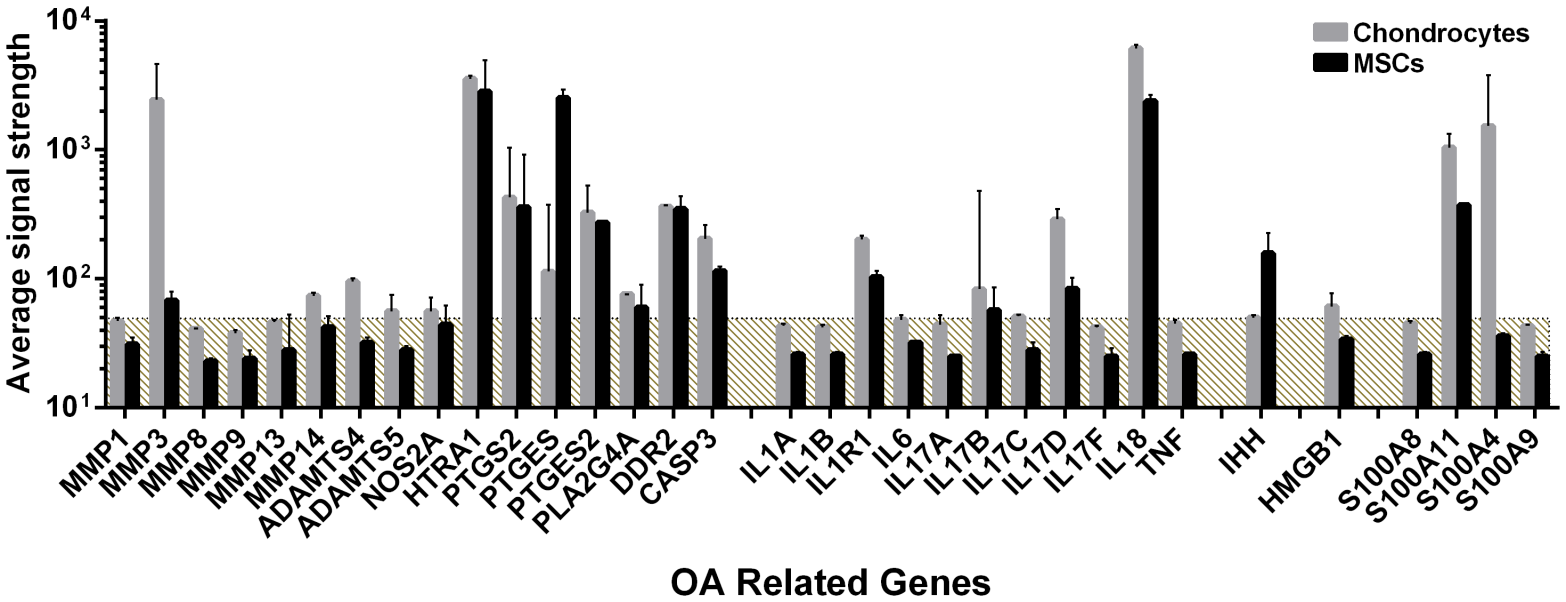
mRNA concentration of selected OA related genes expressed by OA chondrocytes (grey columns) and BM-MSC derived chondrocytes (black columns) on day 21 of differentiation. Expression values are extracted from the whole-genome BeadChip analysis. Each donor data point is the mean value of the Illumina® BeadArray™ Reader scan for that gene and that donor. Columns represent median values for the three donors, and variability whiskers represent range.

As the chondrocytes were harvested from OA joints, and thus could be suspected of expressing genes known to be involved in OA pathogenesis, we identified a large number of genes known to be involved in this disease from the microarray comparison. These were genes encoding enzymes known to be involved in ECM remodeling (*MMPs 1, 3, 8, 9, 13* and *14*, and *ADAMTS4* and *5*), genes encoding enzymes involved in prostaglandin E2 synthesis (*PTGS2, PTGES, PTGES2,* and *PLA2G4A*), genes encoding some other enzymes (*NOS2A, HTRA1, DDR2,* and *CASP3*), genes encoding inflammatory substances (*ILs 1A, 1B, 6, 17A, 17B, 17C, 17D, 17F, 18, TNF*, and *IL1R1*), some genes in the S100A series (*S100A4, A8, A9*,and *A11*), the secreted signaling molecule *IHH* and the damage-associated molecular pattern protein *HMGB1*, all of these genes that have been described in recent reviews on the pathogenesis of OA [21], [37], [38]. The results are shown in Figure 4. Surprisingly, most of these genes were either not expressed or only expressed at levels close to background in any of these cell populations. However, *MMP3, S100A4, ADAMTS4*, and *IL17D* were all expressed at higher levels in the OA chondrocytes, while *PTGES* and *IHH* were expressed at higher levels in the differentiated BM-MSCs. *HTRA1, PTGS2, PTGES2, DDR, CASP3, IL18*, and *S100A11* were expressed at moderate to high levels in both populations.

## Discussion

Tissue engineering of hyaline cartilage by *in vitro* culture of cells in a biomaterial may provide the best treatment option for lesions of articular cartilage. However, in order to tolerate the functional demands put on the cartilage within weight bearing joints over decades, the implant should be as close to native hyaline cartilage as possible, both for the composition and structure of the ECM. OA patients are candidates for treatment with tissue engineered cartilage. Autologous cells, which may be used to produce cartilage *in vitro* for these patients, are articular chondrocytes and MSCs. However, as OA may be a generalized disease within the affected joint, and perhaps also a systemic disease [36], [38], these cell populations may both be affected by the OA disease process. An alternative may be to use allogeneic BM-MSCs from healthy donors. Such cells are easily obtained, as the bone marrow aspiration process carries very limited pain and morbidity, and the cells are easily expanded *in vitro*. Transplantation of allogeneic cells would normally lead to immune rejection of the transplanted cells. However, several observations and facts suggest that this might not occur if allogeneic MSCs are used in scaffolds for treatment of cartilage lesions. First, MSCs have been described to be, at least in part, immunoprivileged cells [39], [40]. Second, MSCs are known to have immunosuppressive properties [25], and this could stop an allogeneic immune response in its early stages. And finally, hyaline cartilage has no blood supply and is, to a minimal degree, in contact with the immune system. A tissue engineered implant entirely within the articular cartilage may, therefore, in effect be within an immunosequestered site. Thus, allogeneic MSCs differentiated to chondrocytes and used in tissue engineering strategies for patients with early OA may represent a viable treatment strategy. On this background, we decided to compare chondrocytes harvested from joints with very advanced OA with BM-MSCs from healthy donors for their ability to act as a cellular substrate for *in vitro* tissue engineering of hyaline cartilage.

COL2 is the most abundant and important of the ECM molecules in cartilage. Using our recently established *in vitro* culture procedure for articular chondrocytes, allowing these cells to expand in their own matrix [27], [41], *COL2A1* mRNA expression was maintained in OA chondrocytes after the *in vitro* expansion, and remained at high levels during chondrogenic differentiation in 3D culture. At the protein level, COL2 was synthesized into the extracellular space around most of the chondrocytes at three weeks. BM-MSCs do not express *COL2A1* mRNA during 2D expansion, but following chondrogenic differentiation in 3D, the *COL2A1* mRNA level increased quickly, and was slightly higher than that observed for the OA chondrocytes at two and three weeks. This difference was not discernible in the less sensitive microarray assay, and there was no obvious difference in COL2 protein synthesis at three weeks. Of other molecules known to be involved in hyaline chondrogenesis, mRNAs for COL9, cartilage intermediate layer protein 1, and decorin were increased in OA chondrocytes by microarray analysis. However, some hyaline cartilage mRNAs, such as *FN1*, *FBLN1*, and *ACAN* were slightly higher in the BM-MSC derived chondrocytes. Notably; mRNAs for most of the other molecules required for synthesis of hyaline cartilage ECM were expressed in both cell types, at similar levels. This suggests that both cell types, embedded in alginate disc scaffolds and exposed to chondrogenic differentiation medium are able to produce the molecules required for synthesis of hyaline cartilage ECM.

Certain molecules, not native to hyaline cartilage, were also expressed in these cells. *COL1A1* encoding the collagen predominantly responsible for the fibrous component of fibrocartilage. This mRNA was highly expressed in both cell types, but more in BM-MSC derived chondrocytes. *COL3A1*, which encodes a protein involved with COL1 in fibrocartilage, but also has a place in hyaline cartilage, was slightly higher in OA chondrocytes. Interestingly, several molecules that are thought to be markers of OA pathogenesis showed higher expression in the differentiated BM-MSCs than in the differentiated OA chondrocytes. The molecule most frequently mentioned in this context is *COL10A1* [42]. *COL10A1* was one of the most differentially expressed molecules between the two cell populations, 85-fold higher in the differentiated BM-MSCs by RT-qPCR. This difference seemed to be reflected at the protein level also, not predominantly by the amount of COL10 synthesized from each cell, but by the proportion of cells synthesizing COL10. Most of the differentiated BM-MSCs synthesized COL10, while <1% of the OA chondrocytes had synthesized this collagen. For the OA chondrocytes, this may represent the cells known to produce COL10 *in vivo* [43], [44]. This could mean that the differentiation cocktail was unable to induce COL10 production in the chondrocytes that did not already produce COL10 *in vivo*. Undifferentiated BM-MSCs also express negligible or no COL10 mRNA [17]. Here, evidently, the epigenetic restrictions to COL10 mRNA and protein synthesis could be overcome by the differentiation cocktail and 3D culture, at least in the majority of the cells. Other molecules known to be markers of chondrocyte hypertrophy during embryological chondrogenesis are *RUNX2, ALPL*, and *MMP13*. Of these, RUNX2 and ALPL were expressed at higher levels in BM-MSC derived chondrocytes, while the expression of MMP13 was very low in both cell populations by RT-qPCR, and not discernible in the microarray assay. Many of the processes associated with chondrocyte hypertrophy, including chondrocyte apoptosis and ECM mineralization have been suggested to also be involved in OA pathogenesis [21], [23], [35], [36], [45]. We have seen no evidence of apoptosis or mineralization occurring during 6–8 week cultures of BM-MSCs exposed to chondrogenic differentiation medium in alginate (A. Küchler, unpublished results), which may suggest that these genetic markers of chondrocyte hypertrophy and OA pathogenesis have no functional correlate during *in vitro* chondrogenesis. However, COL10 is distributed within the extracellular space in these longer term cultures. COL10 is a short, sheet forming collagen [46], unlikely to contribute to tensile strength, and as such not desirable in hyaline ECM.

A number of other genes known to encode proteins involved in OA pathogenesis but not associated with chondrocyte hypertrophy were also investigated. These proteins have been detected in the synovial fluid and in areas of ECM depletion in the cartilage of OA joints. Interestingly, many of these genes were expressed neither in OA chondrocytes nor in BM-MSC derived chondrocytes. This was true particularly for most of the mediators of inflammation known to be associated with OA, but also for most of the enzymes known to degrade hyaline cartilage. One possible explanation for this unexpected finding could be that the OA chondrocytes expressed the OA associated genes at the time of their harvest from the OA cartilage, and subsequently lost expression in the course of *in vitro* expansion and chondrogenic redifferentiation. If so, the fact that the OA genes are no longer expressed may mean that these chondrocytes will not contribute to OA progression when the tissue engineered cartilage is implanted, although this issue can really only be ascertained following *in vivo* implantation. Another possibility could be that the chondrocytes that were used for this *in vitro* chondrogenesis assay, harvested from a healthy-looking part of the OA cartilage, never expressed these genes. This may be the case if the OA-associated proteins were produced by other cells in the vicinity such as the chondrocytes located within the lesion, by cells in the synovial tissue or both. In this situation the cultured OA chondrocytes also most probably would not contribute to progression towards OA after implantation.

Four OA-associated genes were clearly more highly expressed in the cultured OA chondrocytes than in the BM-MSC derived chondrocytes: *MMP3, ADAMTS4, IL17D*, and *S100A4*. *MMP3* encodes a matrix metalloproteinase which degrades fibronectin, laminin, COL3, 4, 9, and 10, and cartilage proteoglycans. Of these, fibronectin, COL9, and proteoglycans, such as ACAN, are important for the normal functioning of articular cartilage. Of these again, only ACAN was investigated at the protein level in the present study and, paradoxically, found to be more widely distributed around the OA chondrocytes. This may suggest that the upregulation of the *MMP3* mRNA in the OA chondrocytes may not have a detrimental effect on cartilage ECM, at least not on ACAN. Complete insight into the effect of MMP3 on articular cartilage would, however, require a more targeted study. *ADAMTS4* was expressed at low levels in OA chondrocytes, but was not expressed at all in the differentiated BM-MSCs. ADAMTS4 is also a metalloproteinase which degrades ACAN. Thus, as with *MMP3*, the observation that more ACAN seemed to be observed around the OA chondrocytes than the BM-MSC derived chondrocytes would suggest that the low-level upregulation of *ADAMTS4* gene expression did not significantly influence ECM synthesis of the OA chondrocytes in alginate discs. *S100A4* encodes a calcium-binding protein. It is best known for its role in cancer metastasis, but has also been found to be upregulated in the synovium of rheumatoid arthritis to a much greater extent than in OA. It is thought to be involved in regulation of several matrix-degrading enzymes, but its exact role in OA remains to be determined [47]. Finally, *IL17D* was also expressed at a higher level in OA chondrocytes than in differentiated BM-MSCs. The encoded cytokine, also known as interleukin (IL) 27, is best known for its ability to induce other inflammatory cytokines from endothelial cells and immune cells. In OA it is thought to be induced by IL-1β and TNFα [37]. However, genes encoding these cytokines were not expressed in either cell subset. Thus, the mechanism responsible for the expression of *IL17D* mRNA and the importance of this expression for the use of OA chondrocytes in tissue engineering of cartilage remains unknown at this time.

Two genes associated with OA, but not directly involved in chondrogenesis, *PTGES* and *IHH,* were expressed at higher levels in differentiated BM-MSCs than in OA chondrocytes. *PTGES* is involved in the synthesis of prostaglandin E2 (PGE2). The gene encoding the first enzyme in this pathway, cytosolic phospholipase A2s (*PLA2G4A*) was expressed at very low levels in both cell types. The gene encoding the subsequent enzyme, cycloxygenase 2 (*PTGS2*), which is normally the rate limiting enzyme, was expressed at moderate levels in both cell types. Finally, prostaglandin E synthase (*PTGES*) was very much more expressed in the differentiated BM-MSCs [48]. PGE2 could potentially be an unwanted molecule produced by implanted cartilage made from BM-MSCs. However, since *PLA2G4A* is only barely expressed in the two cell types, which most likely makes this the rate limiting step, it seems unlikely that the upregulation of *PTGES* will transfer a propensity for OA development through the implanted cartilage. *IHH* has only recently been implicated in the pathogenesis of OA [49]. The mechanism is not fully elucidated, but stimulation of RUNX2 and ADAMTS5 was suggested. In the cells discussed here, ADAMTS5 was expressed at extremely low levels or not at all, while there was a trend for RUNX2 to be expressed at higher levels in the BM-MSC derived chondrocytes by RT-qPCR, perhaps consistent with a stimulatory effect by upregulated IHH.

In conclusion, we show here that both OA chondrocytes and BM-MSCs produce the vast majority of the mRNA molecules required for synthesis of hyaline cartilage when placed in an alginate biomaterial and exposed to chondrogenic differentiation cocktail. For those molecules tested, mRNA expression was translated into protein synthesis. Unfortunately, both cell populations also produced molecules responsible for fibrocartilage. Surprisingly, many of the markers thought to be associated with OA pathogenesis were expressed at higher levels in the differentiated BM-MSCs than in the OA chondrocytes, and the differential synthesis of COL10 may have functional consequences. Karlsson et al, using pellet mass cultures and a restricted number of gene assays, have made similar observations [50]. To make a final decision between these cells, more studies are required to examine other differentiation strategies, other biomaterials, more parallel donors, and longer duration of cell/scaffold constructs *in vitro*. Subsequently, the chosen cell/scaffold constructs should be tested in animal models where OA chondrocytes may be compared with healthy BM-MSCs within the micro-environment, and under the dynamic weight and strain conditions to which native cartilage is exposed *in vivo* [51]–[53]. Eventually, these studies should result in the generation of tissue engineered implants which will provide patients with lesions of articular cartilage with a treatment option that will last for life.

## Supporting Information

Table S1 **Genes upregulated in osteoarthritis (OA) chondrocytes embedded in alginate scaffold with chondrogenic medium after 21 days.**
(DOC)

Table S2 **Genes upregulated in human bone marrow (BM)-MSCs embedded in alginate scaffold with chondrogenic medium after 21 days.**
(DOC)

Table S3 **Gene Ontology comparison results on OA chondrocytes (A) and chondrogenic differentiated human BM-MSCs (B).**
(DOC)
