# Supplementary material for: Similar Properties of Chondrocytes from Osteoarthritis Joints and Mesenchymal Stem Cells from Healthy Donors for Tissue Engineering of Articular Cartilage
Source: PLoS One. 2013 May 9;8(5):e62994. doi: 10.1371/journal.pone.0062994 (PMC3650033; doi:10.1371/journal.pone.0062994)
Supplement: Table S1 — Genes upregulated in osteoarthritis (OA) chondrocytes embedded in alginate scaffold with chondrogenic medium after 21 days. (DOC) [file pone.0062994.s001.doc]

**Supplementary Table S1.** Genes upregulated in osteoarthritis chondrocytes embedded in alginate scaffold with chondrogenic medium after 21 days

| **Rank** | **Symbol** | **DEFINITION** | **Fold change** |
| --- | --- | --- | --- |
| 1 | TPR | Homo sapiens translocated promoter region (to activated MET oncogene) (TPR), mRNA. | 378,8 |
| 2 | CYTL1 | Homo sapiens cytokine-like 1 (CYTL1), mRNA. | 36,8 |
| 3 | S100A4 | Homo sapiens S100 calcium binding protein A4 (S100A4), transcript variant 2, mRNA. | 30,4 |
| 4 | ITM2A | Homo sapiens integral membrane protein 2A (ITM2A), mRNA. | 30,5 |
| 5 | MMP7 | Homo sapiens matrix metallopeptidase 7 (matrilysin, uterine) (MMP7), mRNA. | 25,0 |
| 6 | GPM6B | Homo sapiens glycoprotein M6B (GPM6B), transcript variant 1, mRNA. | 26,2 |
| 7 | MMP7 | Homo sapiens matrix metallopeptidase 7 (matrilysin, uterine) (MMP7), mRNA. | 24,3 |
| 8 | S100A4 | Homo sapiens S100 calcium binding protein A4 (S100A4), transcript variant 2, mRNA. | 25,5 |
| 9 | MMP3 | Homo sapiens matrix metallopeptidase 3 (stromelysin 1, progelatinase) (MMP3), mRNA. | 21,1 |
| 10 | CDO1 | Homo sapiens cysteine dioxygenase, type I (CDO1), mRNA. | 17,9 |
| 11 | C6orf117 | Homo sapiens chromosome 6 open reading frame 117 (C6orf117), mRNA. | 17,2 |
| 12 | H19 | Homo sapiens H19, imprinted maternally expressed transcript (non-protein coding) (H19), non-coding RNA. | 14,9 |
| 13 | GPM6B | Homo sapiens glycoprotein M6B (GPM6B), transcript variant 1, mRNA. | 15,9 |
| 14 | CDO1 | Homo sapiens cysteine dioxygenase, type I (CDO1), mRNA. | 15,0 |
| 15 | TCEAL2 | Homo sapiens transcription elongation factor A (SII)-like 2 (TCEAL2), mRNA. | 14,4 |
| 16 | ANGPTL5 | Homo sapiens angiopoietin-like 5 (ANGPTL5), mRNA. | 13,7 |
| 17 | ABI3BP | Homo sapiens ABI gene family, member 3 (NESH) binding protein (ABI3BP), mRNA. | 13,3 |
| 18 | GDF10 | Homo sapiens growth differentiation factor 10 (GDF10), mRNA. | 13,1 |
| 19 | EPB41L3 | Homo sapiens erythrocyte membrane protein band 4.1-like 3 (EPB41L3), mRNA. | 13,4 |
| 20 | GPR98 | Homo sapiens G protein-coupled receptor 98 (GPR98), transcript variant 1, mRNA. | 13,0 |
| 21 | DPT | Homo sapiens dermatopontin (DPT), mRNA. | 12,5 |
| 22 | C2orf40 | Homo sapiens chromosome 2 open reading frame 40 (C2orf40), mRNA. | 12,0 |
| 23 | GGTA1 | Homo sapiens glycoprotein, alpha-galactosyltransferase 1 (GGTA1), non-coding RNA. | 11,4 |
| 24 | TNFRSF11B | Homo sapiens tumor necrosis factor receptor superfamily, member 11b (TNFRSF11B), mRNA. | 11,0 |
| 25 | MT1M | Homo sapiens metallothionein 1M (MT1M), mRNA. | 10,2 |
| 26 | PDK4 | Homo sapiens pyruvate dehydrogenase kinase, isozyme 4 (PDK4), mRNA. | 10,0 |
| 27 | OGN | Homo sapiens osteoglycin (OGN), transcript variant 3, mRNA. | 9,8 |
| 28 | MMP23B | Homo sapiens matrix metallopeptidase 23B (MMP23B), mRNA. | 8,8 |
| 29 | CRTAC1 | Homo sapiens cartilage acidic protein 1 (CRTAC1), mRNA. | 8,6 |
| 30 | PLA2G2A | Homo sapiens phospholipase A2, group IIA (platelets, synovial fluid) (PLA2G2A), mRNA. | 8,3 |
| 31 | HOPX | Homo sapiens HOP homeobox (HOPX), transcript variant 3, mRNA. | 8,6 |
| 32 |  | Homo sapiens, clone IMAGE:3618365, mRNA | 8,1 |
| 33 | MYOT | Homo sapiens myotilin (MYOT), mRNA. | 8,1 |
| 34 | GPR98 | Homo sapiens G protein-coupled receptor 98 (GPR98), transcript variant 2, non-coding RNA. | 8,1 |
| 35 | GPX3 | Homo sapiens glutathione peroxidase 3 (plasma) (GPX3), mRNA. | 8,2 |
| 36 | AOX1 | Homo sapiens aldehyde oxidase 1 (AOX1), mRNA. | 8,1 |
| 37 | TNC | Homo sapiens tenascin C (hexabrachion) (TNC), mRNA. | 7,7 |
| 38 | SVEP1 | Homo sapiens sushi, von Willebrand factor type A, EGF and pentraxin domain containing 1 (SVEP1), mRNA. | 7,9 |
| 39 | PARM1 | Homo sapiens prostate androgen-regulated mucin-like protein 1 (PARM1), mRNA. | 7,1 |
| 40 | SERPINA1 | Homo sapiens serpin peptidase inhibitor, clade A (alpha-1 antiproteinase, antitrypsin), member 1 (SERPINA1), transcript variant 1, mRNA. | 7,0 |
| 41 | SERPINA1 | Homo sapiens serpin peptidase inhibitor, clade A (alpha-1 antiproteinase, antitrypsin), member 1 (SERPINA1), transcript variant 2, mRNA. | 6,9 |
| 42 | FABP4 | Homo sapiens fatty acid binding protein 4, adipocyte (FABP4), mRNA. | 6,4 |
| 43 | IGFBP6 | Homo sapiens insulin-like growth factor binding protein 6 (IGFBP6), mRNA. | 6,4 |
| 44 | OGN | Homo sapiens osteoglycin (OGN), transcript variant 3, mRNA. | 6,4 |
| 45 | SPINT2 | Homo sapiens serine peptidase inhibitor, Kunitz type, 2 (SPINT2), mRNA. | 6,3 |
| 46 | SLC7A2 | Homo sapiens solute carrier family 7 (cationic amino acid transporter, y+ system), member 2 (SLC7A2), transcript variant 2, mRNA. | 6,6 |
| 47 | CCBP2 | Homo sapiens chemokine binding protein 2 (CCBP2), mRNA. | 6,6 |
| 48 | MFAP4 | Homo sapiens microfibrillar-associated protein 4 (MFAP4), mRNA. | 6,0 |
| 49 | TNC | Homo sapiens tenascin C (TNC), mRNA. | 6,0 |
| 50 | GPR126 | Homo sapiens G protein-coupled receptor 126 (GPR126), transcript variant a2, mRNA. | 5,9 |
| 51 | ZCCHC5 | Homo sapiens zinc finger, CCHC domain containing 5 (ZCCHC5), mRNA. | 5,5 |
| 52 |  | Homo sapiens cDNA FLJ44441 fis, clone UTERU2020242 | 5,7 |
| 53 | OLFM1 | Homo sapiens olfactomedin 1 (OLFM1), transcript variant 1, mRNA. | 5,4 |
| 54 | GALM | Homo sapiens galactose mutarotase (aldose 1-epimerase) (GALM), mRNA. | 5,8 |
| 55 | COL3A1 | Homo sapiens collagen, type III, alpha 1 (COL3A1), mRNA. | 5,7 |
| 56 | GLT8D2 | Homo sapiens glycosyltransferase 8 domain containing 2 (GLT8D2), mRNA. | 5,4 |
| 57 | ADAMTSL3 | Homo sapiens ADAMTS-like 3 (ADAMTSL3), mRNA. | 5,4 |
| 58 |  | in27e08.x1 Human Fetal Pancreas 1B Homo sapiens cDNA clone IMAGE: 3, mRNA sequence | 5,5 |
| 59 | MAMDC2 | Homo sapiens MAM domain containing 2 (MAMDC2), mRNA. | 5,5 |
| 60 | PRKAG1 | Homo sapiens protein kinase, AMP-activated, gamma 1 non-catalytic subunit (PRKAG1), transcript variant 2, mRNA. | 5,1 |
| 61 | SERPINA5 | Homo sapiens serpin peptidase inhibitor, clade A (alpha-1 antiproteinase, antitrypsin), member 5 (SERPINA5), mRNA. | 5,2 |
| 62 | CILP2 | Homo sapiens cartilage intermediate layer protein 2 (CILP2), mRNA. | 5,1 |
| 63 |  | AV681673 GKB Homo sapiens cDNA clone GKBABD06 5, mRNA sequence | 5,0 |
| 64 | KCNIP3 | Homo sapiens Kv channel interacting protein 3, calsenilin (KCNIP3), transcript variant 1, mRNA. | 5,0 |
| 65 | MYO1D | Homo sapiens myosin ID (MYO1D), mRNA. | 5,0 |
| 66 | FBXO2 | Homo sapiens F-box protein 2 (FBXO2), mRNA. | 4,8 |
| 67 | AMTN | Homo sapiens amelotin (AMTN), mRNA. | 6,4 |
| 68 | SLC1A4 | Homo sapiens solute carrier family 1 (glutamate/neutral amino acid transporter), member 4 (SLC1A4), mRNA. | 5,0 |
| 69 | APCDD1 | Homo sapiens adenomatosis polyposis coli down-regulated 1 (APCDD1), mRNA. | 4,7 |
| 70 | SERPINA1 | Homo sapiens serpin peptidase inhibitor, clade A (alpha-1 antiproteinase, antitrypsin), member 1 (SERPINA1), transcript variant 3, mRNA. | 4,9 |
| 71 | MAPK10 | Homo sapiens mitogen-activated protein kinase 10 (MAPK10), transcript variant 2, mRNA. | 4,9 |
| 72 |  | nj54b12.s1 NCI_CGAP_Pr9 Homo sapiens cDNA clone IMAGE:996287, mRNA sequence | 4,5 |
| 73 | NME2 | Homo sapiens non-metastatic cells 2, protein (NM23B) expressed in (NME2), transcript variant 2, mRNA. | 4,6 |
| 74 |  | Homo sapiens mRNA; cDNA DKFZp686F09166 (from clone DKFZp686F09166) | 4,6 |
| 75 | CLIC6 | Homo sapiens chloride intracellular channel 6 (CLIC6), mRNA. | 4,9 |
| 76 | CD302 | Homo sapiens CD302 molecule (CD302), mRNA. | 4,6 |
| 77 | POSTN | Homo sapiens periostin, osteoblast specific factor (POSTN), mRNA. | 4,6 |
| 78 | WISP2 | Homo sapiens WNT1 inducible signaling pathway protein 2 (WISP2), mRNA. | 4,6 |
| 79 | OMD | Homo sapiens osteomodulin (OMD), mRNA. | 4,9 |
| 80 | CRIP1 | Homo sapiens cysteine-rich protein 1 (intestinal) (CRIP1), mRNA. | 4,5 |
| 81 | ATP1B1 | Homo sapiens ATPase, Na+/K+ transporting, beta 1 polypeptide (ATP1B1), transcript variant 1, mRNA. | 4,5 |
| 82 | CD44 | Homo sapiens CD44 molecule (Indian blood group) (CD44), transcript variant 4, mRNA. | 4,6 |
| 83 | ANGPTL7 | Homo sapiens angiopoietin-like 7 (ANGPTL7), mRNA. | 4,7 |
| 84 | RBPMS2 | Homo sapiens RNA binding protein with multiple splicing 2 (RBPMS2), mRNA. | 4,4 |
| 85 | NEXN | Homo sapiens nexilin (F actin binding protein) (NEXN), mRNA. | 4,4 |
| 86 | RASL12 | Homo sapiens RAS-like, family 12 (RASL12), mRNA. | 4,8 |
| 87 | CGNL1 | Homo sapiens cingulin-like 1 (CGNL1), mRNA. | 4,3 |
| 88 | THBS3 | Homo sapiens thrombospondin 3 (THBS3), mRNA. | 4,6 |
| 89 | FBLN5 | Homo sapiens fibulin 5 (FBLN5), mRNA. | 4,2 |
| 90 | FBLN5 | Homo sapiens fibulin 5 (FBLN5), mRNA. | 4,3 |
| 91 | SMOC1 | Homo sapiens SPARC related modular calcium binding 1 (SMOC1), transcript variant 1, mRNA. | 4,3 |
| 92 | RTN1 | Homo sapiens reticulon 1 (RTN1), transcript variant 1, mRNA. | 4,1 |
| 93 | GPM6B | Homo sapiens glycoprotein M6B (GPM6B), transcript variant 4, mRNA. | 4,4 |
| 94 | ELN | Homo sapiens elastin (ELN), transcript variant 4, mRNA. | 4,3 |
| 95 | EFEMP1 | Homo sapiens EGF-containing fibulin-like extracellular matrix protein 1 (EFEMP1), transcript variant 2, mRNA. | 4,2 |
| 96 | FYB | Homo sapiens FYN binding protein (FYB-120/130) (FYB), transcript variant 1, mRNA. | 4,0 |
| 97 | SFRS5 | Homo sapiens splicing factor, arginine/serine-rich 5 (SFRS5), transcript variant 2, mRNA. | 4,0 |
| 98 | IFIT3 | Homo sapiens interferon-induced protein with tetratricopeptide repeats 3 (IFIT3), mRNA. | 3,9 |
| 99 | MTE | Homo sapiens metallothionein E (MTE), mRNA. | 4,1 |
| 100 | ECM2 | Homo sapiens extracellular matrix protein 2, female organ and adipocyte specific (ECM2), mRNA. | 4,0 |
| 101 | RGS5 | Homo sapiens regulator of G-protein signaling 5 (RGS5), mRNA. | 4,3 |
| 102 | RCL1 | Homo sapiens RNA terminal phosphate cyclase-like 1 (RCL1), mRNA. | 3,8 |
| 103 | RPESP | Homo sapiens RPE-spondin (RPESP), mRNA. | 4,0 |
| 104 | MXRA5 | Homo sapiens matrix-remodelling associated 5 (MXRA5), mRNA. | 4,3 |
| 105 | OLFM1 | Homo sapiens olfactomedin 1 (OLFM1), transcript variant 2, mRNA. | 3,9 |
| 106 | GPNMB | Homo sapiens glycoprotein (transmembrane) nmb (GPNMB), transcript variant 1, mRNA. | 3,8 |
| 107 |  | Homo sapiens mRNA; cDNA DKFZp586B0220 (from clone DKFZp586B0220) | 3,8 |
| 108 | KBTBD9 | PREDICTED: Homo sapiens kelch repeat and BTB (POZ) domain containing 9 (KBTBD9), mRNA. | 3,8 |
| 109 | DSC2 | Homo sapiens desmocollin 2 (DSC2), transcript variant Dsc2b, mRNA. | 3,8 |
| 110 | SFRS5 | Homo sapiens splicing factor, arginine/serine-rich 5 (SFRS5), transcript variant 2, mRNA. | 3,8 |
| 111 | GALNT4 | Homo sapiens UDP-N-acetyl-alpha-D-galactosamine:polypeptide N-acetylgalactosaminyltransferase 4 (GalNAc-T4) (GALNT4), mRNA. | 3,7 |
| 112 | PRKAG1 | Homo sapiens protein kinase, AMP-activated, gamma 1 non-catalytic subunit (PRKAG1), transcript variant 1, mRNA. | 3,7 |
| 113 | CXCR7 | Homo sapiens chemokine (C-X-C motif) receptor 7 (CXCR7), mRNA. | 4,2 |
| 114 | HIST2H2BE | Homo sapiens histone cluster 2, H2be (HIST2H2BE), mRNA. | 4,0 |
| 115 | GREM1 | Homo sapiens gremlin 1, cysteine knot superfamily, homolog (Xenopus laevis) (GREM1), mRNA. | 4,2 |
| 116 | LRIG1 | Homo sapiens leucine-rich repeats and immunoglobulin-like domains 1 (LRIG1), mRNA. | 3,7 |
| 117 | C5orf4 | Homo sapiens chromosome 5 open reading frame 4 (C5orf4), mRNA. | 4,4 |
| 118 | ARHGAP20 | Homo sapiens Rho GTPase activating protein 20 (ARHGAP20), mRNA. | 3,8 |
| 119 | SLC14A1 | Homo sapiens solute carrier family 14 (urea transporter), member 1 (Kidd blood group) (SLC14A1), mRNA. | 4,0 |
| 120 | HSPB8 | Homo sapiens heat shock 22kDa protein 8 (HSPB8), mRNA. | 3,8 |
| 121 | PDE4D | Homo sapiens phosphodiesterase 4D, cAMP-specific (phosphodiesterase E3 dunce homolog, Drosophila) (PDE4D), mRNA. | 3,8 |
| 122 | PITX1 | Homo sapiens paired-like homeodomain transcription factor 1 (PITX1), mRNA. | 3,6 |
| 123 | CDH19 | Homo sapiens cadherin 19, type 2 (CDH19), mRNA. | 3,8 |
| 124 | CYP3A5 | Homo sapiens cytochrome P450, family 3, subfamily A, polypeptide 5 (CYP3A5), mRNA. | 4,2 |
| 125 | LOC88523 | Homo sapiens CG016 (LOC88523), mRNA. | 3,7 |
| 126 | HIST2H2AA3 | Homo sapiens histone cluster 2, H2aa3 (HIST2H2AA3), mRNA. | 3,7 |
| 127 |  | Homo sapiens cDNA FLJ31059 fis, clone HSYRA2000832 | 3,7 |
| 128 | LIMCH1 | Homo sapiens LIM and calponin homology domains 1 (LIMCH1), mRNA. | 3,8 |
| 129 | NME5 | Homo sapiens non-metastatic cells 5, protein expressed in (nucleoside-diphosphate kinase) (NME5), mRNA. | 3,6 |
| 130 | CYBRD1 | Homo sapiens cytochrome b reductase 1 (CYBRD1), mRNA. | 3,6 |
| 131 | LMO2 | Homo sapiens LIM domain only 2 (rhombotin-like 1) (LMO2), mRNA. | 3,6 |
| 132 | EFCAB1 | Homo sapiens EF-hand calcium binding domain 1 (EFCAB1), mRNA. | 3,7 |
| 133 | TOP2A | Homo sapiens topoisomerase (DNA) II alpha 170kDa (TOP2A), mRNA. | 3,6 |
| 134 | MX1 | Homo sapiens myxovirus (influenza virus) resistance 1, interferon-inducible protein p78 (mouse) (MX1), mRNA. | 4,0 |
| 135 | ECM2 | Homo sapiens extracellular matrix protein 2, female organ and adipocyte specific (ECM2), mRNA. | 3,7 |
| 136 | C1orf112 | Homo sapiens chromosome 1 open reading frame 112 (C1orf112), mRNA. | 3,5 |
| 137 | MAT2B | Homo sapiens methionine adenosyltransferase II, beta (MAT2B), transcript variant 2, mRNA. | 3,5 |
| 138 | HYAL1 | Homo sapiens hyaluronoglucosaminidase 1 (HYAL1), transcript variant 2, mRNA. | 3,6 |
| 139 | ZFP106 | Homo sapiens zinc finger protein 106 homolog (mouse) (ZFP106), mRNA. | 3,6 |
| 140 | C5 | Homo sapiens complement component 5 (C5), mRNA. | 3,6 |
| 141 | ICA1 | Homo sapiens islet cell autoantigen 1, 69kDa (ICA1), transcript variant 2, mRNA. | 3,6 |
| 142 | MSLN | Homo sapiens mesothelin (MSLN), transcript variant 2, mRNA. | 5,1 |
| 143 | SFRP5 | Homo sapiens secreted frizzled-related protein 5 (SFRP5), mRNA. | 3,5 |
| 144 | SOD2 | Homo sapiens superoxide dismutase 2, mitochondrial (SOD2), nuclear gene encoding mitochondrial protein, transcript variant 2, mRNA. | 3,4 |
| 145 | LRRC16 | Homo sapiens leucine rich repeat containing 16 (LRRC16), mRNA. | 3,4 |
| 146 | ZFP106 | Homo sapiens zinc finger protein 106 homolog (mouse) (ZFP106), mRNA. | 3,5 |
| 147 | CCNB2 | Homo sapiens cyclin B2 (CCNB2), mRNA. | 3,5 |
| 148 | TMEM30B | Homo sapiens transmembrane protein 30B (TMEM30B), mRNA. | 3,5 |
| 149 | CXCR7 | Homo sapiens chemokine (C-X-C motif) receptor 7 (CXCR7), transcript variant 1, mRNA. | 3,8 |
| 150 | MGP | Homo sapiens matrix Gla protein (MGP), mRNA. | 3,9 |
| 151 | ADRB2 | Homo sapiens adrenergic, beta-2-, receptor, surface (ADRB2), mRNA. | 3,4 |
| 152 | C20orf127 | Homo sapiens chromosome 20 open reading frame 127 (C20orf127), mRNA. | 3,7 |
| 153 | HIST2H2AA3 | Homo sapiens histone cluster 2, H2aa3 (HIST2H2AA3), mRNA. | 3,4 |
| 154 | CD44 | Homo sapiens CD44 molecule (Indian blood group) (CD44), transcript variant 5, mRNA. | 3,4 |
| 155 | DCN | Homo sapiens decorin (DCN), transcript variant A1, mRNA. | 3,4 |
| 156 | MAB21L2 | PREDICTED: Homo sapiens mab-21-like 2 (C. elegans) (MAB21L2), mRNA. | 3,5 |
| 157 | C19orf44 | Homo sapiens chromosome 19 open reading frame 44 (C19orf44), mRNA. | 3,4 |
| 158 | C4orf34 | Homo sapiens chromosome 4 open reading frame 34 (C4orf34), mRNA. | 3,2 |
| 159 | SH3D19 | Homo sapiens SH3 domain containing 19 (SH3D19), mRNA. | 3,3 |
| 160 | MAP3K8 | Homo sapiens mitogen-activated protein kinase kinase kinase 8 (MAP3K8), mRNA. | 3,4 |
| 161 | ANGPT1 | Homo sapiens angiopoietin 1 (ANGPT1), mRNA. | 3,2 |
| 162 | KIAA0101 | Homo sapiens KIAA0101 (KIAA0101), transcript variant 1, mRNA. | 3,4 |
| 163 | HMMR | Homo sapiens hyaluronan-mediated motility receptor (RHAMM) (HMMR), transcript variant 2, mRNA. | 3,2 |
| 164 | PON3 | Homo sapiens paraoxonase 3 (PON3), mRNA. | 3,3 |
| 165 | SLC38A4 | Homo sapiens solute carrier family 38, member 4 (SLC38A4), mRNA. | 3,5 |
| 166 | LOC646588 | PREDICTED: Homo sapiens hypothetical protein LOC646588 (LOC646588), mRNA. | 3,4 |
| 167 | LRRN3 | Homo sapiens leucine rich repeat neuronal 3 (LRRN3), transcript variant 1, mRNA. | 3,4 |
| 168 | SIPA1L2 | Homo sapiens signal-induced proliferation-associated 1 like 2 (SIPA1L2), mRNA. | 3,3 |
| 169 | CFH | Homo sapiens complement factor H (CFH), transcript variant 2, mRNA. | 3,3 |
| 170 | TNFAIP8 | Homo sapiens tumor necrosis factor, alpha-induced protein 8 (TNFAIP8), transcript variant 2, mRNA. | 3,4 |
| 171 | LOC285929 | PREDICTED: Homo sapiens similar to matrilin 2 precursor, transcript variant 1 (LOC285929), mRNA. | 3,2 |
| 172 | PDGFD | Homo sapiens platelet derived growth factor D (PDGFD), transcript variant 2, mRNA. | 3,3 |
| 173 | NUSAP1 | Homo sapiens nucleolar and spindle associated protein 1 (NUSAP1), transcript variant 2, mRNA. | 3,2 |
| 174 | KIAA1618 | Homo sapiens KIAA1618 (KIAA1618), mRNA. | 3,1 |
| 175 | FAM134B | Homo sapiens family with sequence similarity 134, member B (FAM134B), transcript variant 1, mRNA. | 3,6 |
| 176 | DSC3 | Homo sapiens desmocollin 3 (DSC3), transcript variant Dsc3b, mRNA. | 3,1 |
| 177 | HIST2H2AC | Homo sapiens histone cluster 2, H2ac (HIST2H2AC), mRNA. | 3,2 |
| 178 | CFH | Homo sapiens complement factor H (CFH), transcript variant 2, mRNA. | 3,3 |
| 179 | RUNX1T1 | Homo sapiens runt-related transcription factor 1; translocated to, 1 (cyclin D-related) (RUNX1T1), transcript variant 1, mRNA. | 3,1 |
| 180 | ARHGDIB | Homo sapiens Rho GDP dissociation inhibitor (GDI) beta (ARHGDIB), mRNA. | 3,4 |
| 181 | FAP | Homo sapiens fibroblast activation protein, alpha (FAP), mRNA. | 3,1 |
| 182 | PDGFD | Homo sapiens platelet derived growth factor D (PDGFD), transcript variant 1, mRNA. | 3,2 |
| 183 | CCL2 | Homo sapiens chemokine (C-C motif) ligand 2 (CCL2), mRNA. | 3,3 |
| 184 | ZCCHC5 | Homo sapiens zinc finger, CCHC domain containing 5 (ZCCHC5), mRNA. | 3,1 |
| 185 | MGP | Homo sapiens matrix Gla protein (MGP), mRNA. | 3,5 |
| 186 | PTTG1 | Homo sapiens pituitary tumor-transforming 1 (PTTG1), mRNA. | 3,1 |
| 187 | NOX4 | Homo sapiens NADPH oxidase 4 (NOX4), mRNA. | 3,5 |
| 188 | MT1G | Homo sapiens metallothionein 1G (MT1G), mRNA. | 3,3 |
| 189 | CYBRD1 | Homo sapiens cytochrome b reductase 1 (CYBRD1), mRNA. | 3,0 |
| 190 | CLTB | Homo sapiens clathrin, light chain (Lcb) (CLTB), transcript variant 2, mRNA. | 3,1 |
| 191 | RECK | Homo sapiens reversion-inducing-cysteine-rich protein with kazal motifs (RECK), mRNA. | 3,0 |
| 192 | PXMP2 | Homo sapiens peroxisomal membrane protein 2, 22kDa (PXMP2), mRNA. | 3,0 |
| 193 | SOD2 | Homo sapiens superoxide dismutase 2, mitochondrial (SOD2), nuclear gene encoding mitochondrial protein, transcript variant 3, mRNA. | 3,0 |
| 194 | FNDC1 | Homo sapiens fibronectin type III domain containing 1 (FNDC1), mRNA. | 3,6 |
| 195 | MT1E | Homo sapiens metallothionein 1E (functional) (MT1E), mRNA. | 3,5 |
| 196 | CYSLTR1 | Homo sapiens cysteinyl leukotriene receptor 1 (CYSLTR1), mRNA. | 3,2 |
| 197 | ITGA1 | Homo sapiens integrin, alpha 1 (ITGA1), mRNA. | 3,0 |
| 198 | BFSP1 | Homo sapiens beaded filament structural protein 1, filensin (BFSP1), mRNA. | 3,1 |
| 199 | DEFB1 | Homo sapiens defensin, beta 1 (DEFB1), mRNA. | 3,0 |
| 200 | PIK3R1 | Homo sapiens phosphoinositide-3-kinase, regulatory subunit 1 (alpha) (PIK3R1), transcript variant 1, mRNA. | 3,1 |
| 201 | LOC375295 | PREDICTED: Homo sapiens hypothetical gene supported by BC013438 (LOC375295), mRNA. | 3,1 |
| 202 | SLC9A3R1 | Homo sapiens solute carrier family 9 (sodium/hydrogen exchanger), member 3 regulator 1 (SLC9A3R1), mRNA. | 2,9 |
| 203 | CDKN2C | Homo sapiens cyclin-dependent kinase inhibitor 2C (p18, inhibits CDK4) (CDKN2C), transcript variant 2, mRNA. | 3,0 |
| 204 | GAS7 | Homo sapiens growth arrest-specific 7 (GAS7), transcript variant c, mRNA. | 3,3 |
| 205 | DMKN | Homo sapiens dermokine (DMKN), transcript variant 2, mRNA. | 2,9 |
| 206 | EGR2 | Homo sapiens early growth response 2 (Krox-20 homolog, Drosophila) (EGR2), mRNA. | 3,2 |
| 207 | AHNAK | Homo sapiens AHNAK nucleoprotein (AHNAK), transcript variant 1, mRNA. | 2,9 |
| 208 | CHAD | Homo sapiens chondroadherin (CHAD), mRNA. | 3,1 |
| 209 | LBH | Homo sapiens limb bud and heart development homolog (mouse) (LBH), mRNA. | 2,9 |
| 210 | SORBS1 | Homo sapiens sorbin and SH3 domain containing 1 (SORBS1), transcript variant 3, mRNA. | 2,9 |
| 211 | CILP | Homo sapiens cartilage intermediate layer protein, nucleotide pyrophosphohydrolase (CILP), mRNA. | 2,9 |
| 212 | VIPR1 | Homo sapiens vasoactive intestinal peptide receptor 1 (VIPR1), mRNA. | 3,2 |
| 213 | CDC20 | Homo sapiens cell division cycle 20 homolog (S. cerevisiae) (CDC20), mRNA. | 3,1 |
| 214 | HSPA2 | Homo sapiens heat shock 70kDa protein 2 (HSPA2), mRNA. | 3,0 |
| 215 | LOC647346 | PREDICTED: Homo sapiens similar to Alcohol dehydrogenase class 3 chi chain (Alcohol dehydrogenase class III chi chain) (S-(hydroxymethyl)glutathione dehydrogenase) (Glutathione-dependent formaldehyde dehydrogenase) (FDH), transcript variant 1 (LOC647346), mRNA. | 2,8 |
| 216 | DMKN | Homo sapiens dermokine (DMKN), transcript variant 2, mRNA. | 2,9 |
| 217 | CD79B | Homo sapiens CD79b molecule, immunoglobulin-associated beta (CD79B), transcript variant 3, mRNA. | 3,1 |
| 218 | BARX1 | Homo sapiens BARX homeobox 1 (BARX1), mRNA. | 2,9 |
| 219 | NFIB | Homo sapiens nuclear factor I/B (NFIB), mRNA. | 2,9 |
| 220 | CA2 | Homo sapiens carbonic anhydrase II (CA2), mRNA. | 3,7 |
| 221 | CDC2 | Homo sapiens cell division cycle 2, G1 to S and G2 to M (CDC2), transcript variant 1, mRNA. | 2,8 |
| 222 | HSPA2 | Homo sapiens heat shock 70kDa protein 2 (HSPA2), mRNA. | 3,0 |
| 223 | ELMO1 | Homo sapiens engulfment and cell motility 1 (ELMO1), transcript variant 1, mRNA. | 2,9 |
| 224 | STOM | Homo sapiens stomatin (STOM), transcript variant 1, mRNA. | 2,9 |
| 225 | TMSB4X | Homo sapiens thymosin, beta 4, X-linked (TMSB4X), mRNA. | 2,9 |
| 226 | LAMA2 | Homo sapiens laminin, alpha 2 (LAMA2), transcript variant 2, mRNA. | 2,9 |
| 227 | APPL2 | Homo sapiens adaptor protein, phosphotyrosine interaction, PH domain and leucine zipper containing 2 (APPL2), mRNA. | 2,8 |
| 228 | STK32B | Homo sapiens serine/threonine kinase 32B (STK32B), mRNA. | 3,0 |
| 229 | ALDH1L1 | Homo sapiens aldehyde dehydrogenase 1 family, member L1 (ALDH1L1), mRNA. | 3,0 |
| 230 | EFEMP1 | Homo sapiens EGF-containing fibulin-like extracellular matrix protein 1 (EFEMP1), transcript variant 1, mRNA. | 2,8 |
| 231 | NCAPD3 | Homo sapiens non-SMC condensin II complex, subunit D3 (NCAPD3), mRNA. | 2,9 |
| 232 | RECK | Homo sapiens reversion-inducing-cysteine-rich protein with kazal motifs (RECK), mRNA. | 2,8 |
| 233 | SLPI | Homo sapiens secretory leukocyte peptidase inhibitor (SLPI), mRNA. | 2,8 |
| 234 | TGFBR2 | Homo sapiens transforming growth factor, beta receptor II (70/80kDa) (TGFBR2), transcript variant 1, mRNA. | 2,8 |
| 235 | ACADL | Homo sapiens acyl-Coenzyme A dehydrogenase, long chain (ACADL), nuclear gene encoding mitochondrial protein, mRNA. | 2,8 |
| 236 | MMP23A | Homo sapiens matrix metallopeptidase 23A (MMP23A), mRNA. | 2,8 |
| 237 | KBTBD11 | Homo sapiens kelch repeat and BTB (POZ) domain containing 11 (KBTBD11), mRNA. | 2,8 |
| 238 | TGFBR3 | Homo sapiens transforming growth factor, beta receptor III (TGFBR3), mRNA. | 2,8 |
| 239 | MAPK10 | Homo sapiens mitogen-activated protein kinase 10 (MAPK10), transcript variant 3, mRNA. | 2,8 |
| 240 | VAMP8 | Homo sapiens vesicle-associated membrane protein 8 (endobrevin) (VAMP8), mRNA. | 2,8 |
| 241 | CKS2 | Homo sapiens CDC28 protein kinase regulatory subunit 2 (CKS2), mRNA. | 2,7 |
| 242 | RBM3 | Homo sapiens RNA binding motif (RNP1, RRM) protein 3 (RBM3), mRNA. | 2,7 |
| 243 | SUSD2 | Homo sapiens sushi domain containing 2 (SUSD2), mRNA. | 2,7 |
| 244 | DMKN | Homo sapiens dermokine (DMKN), transcript variant 1, mRNA. | 2,7 |
| 245 | CLCNKA | Homo sapiens chloride channel Ka (CLCNKA), transcript variant 2, mRNA. | 2,8 |
| 246 | IFIT3 | Homo sapiens interferon-induced protein with tetratricopeptide repeats 3 (IFIT3), mRNA. | 2,8 |
| 247 | FLJ10986 | Homo sapiens hypothetical protein FLJ10986 (FLJ10986), mRNA. | 2,7 |
| 248 | ANKRD35 | Homo sapiens ankyrin repeat domain 35 (ANKRD35), mRNA. | 2,7 |
| 249 | GPNMB | Homo sapiens glycoprotein (transmembrane) nmb (GPNMB), transcript variant 2, mRNA. | 2,7 |
| 250 | CCNG2 | Homo sapiens cyclin G2 (CCNG2), mRNA. | 2,7 |
| 251 | C10orf65 | Homo sapiens chromosome 10 open reading frame 65 (C10orf65), mRNA. | 3,0 |
| 252 | C10orf107 | Homo sapiens chromosome 10 open reading frame 107 (C10orf107), mRNA. | 2,9 |
| 253 | FGD5 | Homo sapiens FYVE, RhoGEF and PH domain containing 5 (FGD5), mRNA. | 2,8 |
| 254 | LHFP | Homo sapiens lipoma HMGIC fusion partner (LHFP), mRNA. | 2,7 |
| 255 |  | Homo sapiens cDNA clone IMAGE:5262734 | 2,7 |
| 256 | FLJ22662 | Homo sapiens hypothetical protein FLJ22662 (FLJ22662), mRNA. | 2,7 |
| 257 | BEXL1 | PREDICTED: Homo sapiens brain expressed X-linked-like 1 (BEXL1), mRNA. | 2,8 |
| 258 |  | Homo sapiens cDNA FLJ34736 fis, clone MESAN2008222 | 2,8 |
| 259 | CHD5 | Homo sapiens chromodomain helicase DNA binding protein 5 (CHD5), mRNA. | 2,8 |
| 260 | EFEMP1 | Homo sapiens EGF-containing fibulin-like extracellular matrix protein 1 (EFEMP1), transcript variant 1, mRNA. | 2,7 |
| 261 | ASPM | Homo sapiens asp (abnormal spindle) homolog, microcephaly associated (Drosophila) (ASPM), mRNA. | 2,7 |
| 262 | PYCARD | Homo sapiens PYD and CARD domain containing (PYCARD), transcript variant 1, mRNA. | 2,8 |
| 263 | SCNN1A | Homo sapiens sodium channel, nonvoltage-gated 1 alpha (SCNN1A), mRNA. | 2,8 |
| 264 | CFH | Homo sapiens complement factor H (CFH), transcript variant 2, mRNA. | 2,7 |
| 265 | MDFIC | Homo sapiens MyoD family inhibitor domain containing (MDFIC), mRNA. | 2,6 |
| 266 | IGSF3 | Homo sapiens immunoglobulin superfamily, member 3 (IGSF3), transcript variant 1, mRNA. | 2,6 |
| 267 | BMP4 | Homo sapiens bone morphogenetic protein 4 (BMP4), transcript variant 3, mRNA. | 2,7 |
| 268 | HSD17B14 | Homo sapiens hydroxysteroid (17-beta) dehydrogenase 14 (HSD17B14), mRNA. | 2,7 |
| 269 | PLXNA2 | Homo sapiens plexin A2 (PLXNA2), mRNA. | 2,7 |
| 270 | MGC24103 | PREDICTED: Homo sapiens hypothetical protein MGC24103 (MGC24103), misc RNA. | 2,7 |
| 271 | GCNT1 | Homo sapiens glucosaminyl (N-acetyl) transferase 1, core 2 (beta-1,6-N-acetylglucosaminyltransferase) (GCNT1), transcript variant 4, mRNA. | 2,7 |
| 272 | SMARCD2 | Homo sapiens SWI/SNF related, matrix associated, actin dependent regulator of chromatin, subfamily d, member 2 (SMARCD2), mRNA. | 2,6 |
| 273 | C18orf1 | Homo sapiens chromosome 18 open reading frame 1 (C18orf1), transcript variant b2, mRNA. | 2,7 |
| 274 | ALS2CR4 | Homo sapiens amyotrophic lateral sclerosis 2 (juvenile) chromosome region, candidate 4 (ALS2CR4), transcript variant 1, mRNA. | 2,6 |
| 275 | CCDC28B | Homo sapiens coiled-coil domain containing 28B (CCDC28B), mRNA. | 2,6 |
| 276 | KIF20B | Homo sapiens kinesin family member 20B (KIF20B), mRNA. | 2,6 |
| 277 | ITSN1 | Homo sapiens intersectin 1 (SH3 domain protein) (ITSN1), transcript variant 2, mRNA. | 2,6 |
| 278 | RASSF5 | Homo sapiens Ras association (RalGDS/AF-6) domain family member 5 (RASSF5), transcript variant 2, mRNA. | 2,7 |
| 279 | DSG2 | Homo sapiens desmoglein 2 (DSG2), mRNA. | 2,7 |
| 280 | CLIC3 | Homo sapiens chloride intracellular channel 3 (CLIC3), mRNA. | 2,7 |
| 281 | CENPF | Homo sapiens centromere protein F, 350/400ka (mitosin) (CENPF), mRNA. | 2,6 |
| 282 | SLC7A8 | Homo sapiens solute carrier family 7 (cationic amino acid transporter, y+ system), member 8 (SLC7A8), transcript variant 2, mRNA. | 2,6 |
| 283 | ATP8B2 | Homo sapiens ATPase, class I, type 8B, member 2 (ATP8B2), transcript variant 1, mRNA. | 2,6 |
| 284 | ISM1 | Homo sapiens isthmin 1 homolog (zebrafish) (ISM1), mRNA. | 2,7 |
| 285 | MT1E | Homo sapiens metallothionein 1E (MT1E), mRNA. | 2,8 |
| 286 | GOLSYN | Homo sapiens Golgi-localized protein (GOLSYN), transcript variant 7, mRNA. | 3,0 |
| 287 | CYB5R2 | Homo sapiens cytochrome b5 reductase 2 (CYB5R2), mRNA. | 2,6 |
| 288 | FNDC1 | Homo sapiens fibronectin type III domain containing 1 (FNDC1), mRNA. | 3,0 |
| 289 | UHRF1 | Homo sapiens ubiquitin-like with PHD and ring finger domains 1 (UHRF1), transcript variant 1, mRNA. | 2,6 |
| 290 | PXMP2 | Homo sapiens peroxisomal membrane protein 2, 22kDa (PXMP2), mRNA. | 2,6 |
| 291 | C21orf57 | Homo sapiens chromosome 21 open reading frame 57 (C21orf57), transcript variant 1, mRNA. | 2,6 |
| 292 | AP1S2 | Homo sapiens adaptor-related protein complex 1, sigma 2 subunit (AP1S2), mRNA. | 2,5 |
| 293 | DSG3 | Homo sapiens desmoglein 3 (pemphigus vulgaris antigen) (DSG3), mRNA. | 2,6 |
| 294 | IFT57 | Homo sapiens intraflagellar transport 57 homolog (Chlamydomonas) (IFT57), mRNA. | 2,5 |
| 295 | NICN1 | Homo sapiens nicolin 1 (NICN1), mRNA. | 2,6 |
| 296 | IFIT1 | Homo sapiens interferon-induced protein with tetratricopeptide repeats 1 (IFIT1), transcript variant 2, mRNA. | 2,6 |
| 297 | JAG1 | Homo sapiens jagged 1 (Alagille syndrome) (JAG1), mRNA. | 2,6 |
| 298 | CTTN | Homo sapiens cortactin (CTTN), transcript variant 2, mRNA. | 2,5 |
| 299 | CHEK2 | Homo sapiens CHK2 checkpoint homolog (S. pombe) (CHEK2), transcript variant 1, mRNA. | 2,5 |
| 300 | SNF8 | Homo sapiens SNF8, ESCRT-II complex subunit, homolog (S. cerevisiae) (SNF8), mRNA. | 2,5 |
| 301 | 04.sep | Homo sapiens septin 4 (SEPT4), transcript variant 2, mRNA. | 3,3 |
| 302 | CCNA2 | Homo sapiens cyclin A2 (CCNA2), mRNA. | 2,5 |
| 303 | FAM134B | Homo sapiens family with sequence similarity 134, member B (FAM134B), transcript variant 1, mRNA. | 2,6 |
| 304 | ALPK2 | Homo sapiens alpha-kinase 2 (ALPK2), mRNA. | 3,1 |
| 305 | SMOC2 | Homo sapiens SPARC related modular calcium binding 2 (SMOC2), mRNA. | 2,8 |
| 306 | DBI | Homo sapiens diazepam binding inhibitor (GABA receptor modulator, acyl-Coenzyme A binding protein) (DBI), mRNA. | 2,5 |
| 307 | ANGPT1 | Homo sapiens angiopoietin 1 (ANGPT1), mRNA. | 2,5 |
| 308 | RGS5 | Homo sapiens regulator of G-protein signaling 5 (RGS5), mRNA. | 2,8 |
| 309 | CAV1 | Homo sapiens caveolin 1, caveolae protein, 22kDa (CAV1), mRNA. | 2,6 |
| 310 | CCNG2 | Homo sapiens cyclin G2 (CCNG2), mRNA. | 2,6 |
| 311 | COL24A1 | Homo sapiens collagen, type XXIV, alpha 1 (COL24A1), mRNA. | 2,5 |
| 312 | HILS1 | Homo sapiens histone linker H1 domain, spermatid-specific 1 (HILS1), mRNA. | 2,5 |
| 313 | PLXDC2 | Homo sapiens plexin domain containing 2 (PLXDC2), mRNA. | 2,5 |
| 314 | LBH | PREDICTED: Homo sapiens hypothetical protein DKFZp566J091 (LBH), mRNA. | 2,5 |
| 315 | NBN | Homo sapiens nibrin (NBN), mRNA. | 2,5 |
| 316 | GRK5 | Homo sapiens G protein-coupled receptor kinase 5 (GRK5), mRNA. | 2,6 |
| 317 |  | BX105899 NCI_CGAP_Pr28 Homo sapiens cDNA clone IMAGp998H075756, mRNA sequence | 2,5 |
| 318 |  | Homo sapiens cDNA FLJ37828 fis, clone BRSSN2006575 | 2,5 |
| 319 | C5orf5 | Homo sapiens chromosome 5 open reading frame 5 (C5orf5), mRNA. | 2,5 |
| 320 | MT1H | Homo sapiens metallothionein 1H (MT1H), mRNA. | 2,7 |
| 321 | PDE1A | Homo sapiens phosphodiesterase 1A, calmodulin-dependent (PDE1A), transcript variant 2, mRNA. | 2,5 |
| 322 |  | Homo sapiens mRNA; cDNA DKFZp686H20120 (from clone DKFZp686H20120) | 2,6 |
| 323 | CLCNKA | Homo sapiens chloride channel Ka (CLCNKA), transcript variant 1, mRNA. | 2,5 |
| 324 | TDRD9 | Homo sapiens tudor domain containing 9 (TDRD9), mRNA. | 2,6 |
| 325 | TGFBR2 | Homo sapiens transforming growth factor, beta receptor II (70/80kDa) (TGFBR2), transcript variant 1, mRNA. | 2,5 |
| 326 | AKAP13 | Homo sapiens A kinase (PRKA) anchor protein 13 (AKAP13), transcript variant 2, mRNA. | 2,6 |
| 327 | FAM111A | Homo sapiens family with sequence similarity 111, member A (FAM111A), transcript variant 1, mRNA. | 2,5 |
| 328 | STOM | Homo sapiens stomatin (STOM), transcript variant 1, mRNA. | 2,5 |
| 329 | AP1S2 | Homo sapiens adaptor-related protein complex 1, sigma 2 subunit (AP1S2), mRNA. | 2,4 |
| 330 | LOC644662 | PREDICTED: Homo sapiens hypothetical LOC644662, transcript variant 2 (LOC644662), mRNA. | 2,7 |
| 331 | PRRG4 | Homo sapiens proline rich Gla (G-carboxyglutamic acid) 4 (transmembrane) (PRRG4), mRNA. | 2,6 |
| 332 | STMN1 | Homo sapiens stathmin 1 (STMN1), transcript variant 1, mRNA. | 2,5 |
| 333 | BIRC5 | Homo sapiens baculoviral IAP repeat-containing 5 (BIRC5), transcript variant 1, mRNA. | 2,5 |
| 334 | RRAGB | Homo sapiens Ras-related GTP binding B (RRAGB), transcript variant RAGBs, mRNA. | 2,4 |
| 335 | FXYD6 | Homo sapiens FXYD domain containing ion transport regulator 6 (FXYD6), mRNA. | 2,5 |
| 336 | ID1 | Homo sapiens inhibitor of DNA binding 1, dominant negative helix-loop-helix protein (ID1), transcript variant 2, mRNA. | 2,5 |
| 337 | CD47 | Homo sapiens CD47 molecule (CD47), transcript variant 2, mRNA. | 2,4 |
| 338 | XAF1 | Homo sapiens XIAP associated factor 1 (XAF1), transcript variant 2, mRNA. | 2,6 |
| 339 | SYNM | Homo sapiens synemin, intermediate filament protein (SYNM), transcript variant B, mRNA. | 2,4 |
| 340 | C8orf46 | Homo sapiens chromosome 8 open reading frame 46 (C8orf46), mRNA. | 2,5 |
| 341 | GADD45G | Homo sapiens growth arrest and DNA-damage-inducible, gamma (GADD45G), mRNA. | 2,5 |
| 342 | LYSMD2 | Homo sapiens LysM, putative peptidoglycan-binding, domain containing 2 (LYSMD2), mRNA. | 2,4 |
| 343 | CKS2 | Homo sapiens CDC28 protein kinase regulatory subunit 2 (CKS2), mRNA. | 2,4 |
| 344 | PRKCZ | Homo sapiens protein kinase C, zeta (PRKCZ), transcript variant 1, mRNA. | 2,5 |
| 345 | RAMP1 | Homo sapiens receptor (G protein-coupled) activity modifying protein 1 (RAMP1), mRNA. | 2,5 |
| 346 | RANBP3L | Homo sapiens RAN binding protein 3-like (RANBP3L), mRNA. | 2,5 |
| 347 | COL9A1 | Homo sapiens collagen, type IX, alpha 1 (COL9A1), transcript variant 1, mRNA. | 2,8 |
| 348 | UBA7 | Homo sapiens ubiquitin-like modifier activating enzyme 7 (UBA7), mRNA. | 2,5 |
| 349 | PNPLA7 | Homo sapiens patatin-like phospholipase domain containing 7 (PNPLA7), mRNA. | 2,8 |
| 350 | KIF20A | Homo sapiens kinesin family member 20A (KIF20A), mRNA. | 2,5 |
| 351 | LAMA2 | Homo sapiens laminin, alpha 2 (LAMA2), transcript variant 1, mRNA. | 2,5 |
| 352 | C7orf41 | Homo sapiens chromosome 7 open reading frame 41 (C7orf41), mRNA. | 2,5 |
| 353 | LBH | Homo sapiens limb bud and heart development homolog (mouse) (LBH), mRNA. | 2,4 |
| 354 | SIRPA | Homo sapiens signal-regulatory protein alpha (SIRPA), transcript variant 2, mRNA. | 2,4 |
| 355 | GSTM3 | Homo sapiens glutathione S-transferase M3 (brain) (GSTM3), mRNA. | 2,4 |
| 356 | JAK2 | Homo sapiens Janus kinase 2 (a protein tyrosine kinase) (JAK2), mRNA. | 2,5 |
| 357 | COL16A1 | Homo sapiens collagen, type XVI, alpha 1 (COL16A1), mRNA. | 2,5 |
| 358 | CHEK2 | Homo sapiens CHK2 checkpoint homolog (S. pombe) (CHEK2), transcript variant 1, mRNA. | 2,4 |
| 359 | FST | Homo sapiens follistatin (FST), transcript variant FST344, mRNA. | 2,5 |
| 360 | NET1 | Homo sapiens neuroepithelial cell transforming 1 (NET1), transcript variant 1, mRNA. | 2,5 |
| 361 | CCDC102A | Homo sapiens coiled-coil domain containing 102A (CCDC102A), mRNA. | 2,7 |
| 362 | ZW10 | Homo sapiens ZW10, kinetochore associated, homolog (Drosophila) (ZW10), mRNA. | 2,3 |
| 363 |  | Homo sapiens clone 24583 mRNA sequence | 2,3 |
| 364 | EGR1 | Homo sapiens early growth response 1 (EGR1), mRNA. | 2,4 |
| 365 | AMT | Homo sapiens aminomethyltransferase (AMT), mRNA. | 2,5 |
| 366 | C4orf34 | Homo sapiens chromosome 4 open reading frame 34 (C4orf34), mRNA. | 2,3 |
| 367 | PBK | Homo sapiens PDZ binding kinase (PBK), mRNA. | 2,4 |
| 368 | CFB | Homo sapiens complement factor B (CFB), mRNA. | 2,4 |
| 369 | CHI3L2 | Homo sapiens chitinase 3-like 2 (CHI3L2), transcript variant 1, mRNA. | 2,5 |
| 370 | RPS4Y1 | Homo sapiens ribosomal protein S4, Y-linked 1 (RPS4Y1), mRNA. | 3,0 |
| 371 | CDCA7 | Homo sapiens cell division cycle associated 7 (CDCA7), transcript variant 1, mRNA. | 2,4 |
| 372 | CYP4V2 | Homo sapiens cytochrome P450, family 4, subfamily V, polypeptide 2 (CYP4V2), mRNA. | 2,4 |
| 373 | IQCA1 | Homo sapiens IQ motif containing with AAA domain 1 (IQCA1), mRNA. | 2,4 |
| 374 | SCUBE2 | Homo sapiens signal peptide, CUB domain, EGF-like 2 (SCUBE2), mRNA. | 2,6 |
| 375 | LYL1 | Homo sapiens lymphoblastic leukemia derived sequence 1 (LYL1), mRNA. | 2,3 |
| 376 | TNRC6B | Homo sapiens trinucleotide repeat containing 6B (TNRC6B), transcript variant 2, mRNA. | 2,3 |
| 377 | NET1 | Homo sapiens neuroepithelial cell transforming 1 (NET1), transcript variant 2, mRNA. | 2,6 |
| 378 | METTL7A | Homo sapiens methyltransferase like 7A (METTL7A), mRNA. | 2,4 |
| 379 | LOC388681 | PREDICTED: Homo sapiens similar to phosphodiesterase 4D interacting protein isoform 1 (LOC388681), mRNA. | 2,6 |
| 380 | LOC727935 | PREDICTED: Homo sapiens similar to CHRNA7 (cholinergic receptor, nicotinic, alpha 7, exons 5-10) and FAM7A (family with sequence similarity 7A, exons A-E) fusion (LOC727935), mRNA. | 2,3 |
| 381 | CPXM2 | Homo sapiens carboxypeptidase X (M14 family), member 2 (CPXM2), mRNA. | 2,3 |
| 382 | PAFAH1B1 | Homo sapiens platelet-activating factor acetylhydrolase, isoform Ib, alpha subunit 45kDa (PAFAH1B1), mRNA. | 2,3 |
| 383 | LOC646990 | PREDICTED: Homo sapiens hypothetical protein LOC646990 (LOC646990), mRNA. | 2,4 |
| 384 | C2orf64 | Homo sapiens chromosome 2 open reading frame 64 (C2orf64), mRNA. | 2,3 |
| 385 | UBE2C | Homo sapiens ubiquitin-conjugating enzyme E2C (UBE2C), transcript variant 3, mRNA. | 2,4 |
| 386 | FNIP1 | Homo sapiens folliculin interacting protein 1 (FNIP1), transcript variant 2, mRNA. | 2,3 |
| 387 | LOC645968 | PREDICTED: Homo sapiens similar to 40S ribosomal protein S3a (V-fos transformation effector protein) (LOC645968), mRNA. | 2,3 |
| 388 | KIAA1009 | Homo sapiens KIAA1009 (KIAA1009), mRNA. | 2,3 |
| 389 | PLCE1 | Homo sapiens phospholipase C, epsilon 1 (PLCE1), transcript variant 1, mRNA. | 2,5 |
| 390 | ECHDC2 | Homo sapiens enoyl Coenzyme A hydratase domain containing 2 (ECHDC2), mRNA. | 2,4 |
| 391 | RSU1 | Homo sapiens Ras suppressor protein 1 (RSU1), transcript variant 1, mRNA. | 2,3 |
| 392 | AHNAK | Homo sapiens AHNAK nucleoprotein (AHNAK), transcript variant 2, mRNA. | 2,3 |
| 393 | COL9A1 | Homo sapiens collagen, type IX, alpha 1 (COL9A1), transcript variant 1, mRNA. | 2,5 |
| 394 | PTTG1 | Homo sapiens pituitary tumor-transforming 1 (PTTG1), mRNA. | 2,3 |
| 395 | ARL9 | Homo sapiens ADP-ribosylation factor-like 9 (ARL9), mRNA. | 2,4 |
| 396 | UST | Homo sapiens uronyl-2-sulfotransferase (UST), mRNA. | 2,3 |
| 397 | SELENBP1 | Homo sapiens selenium binding protein 1 (SELENBP1), mRNA. | 2,6 |
| 398 | PRKCDBP | Homo sapiens protein kinase C, delta binding protein (PRKCDBP), mRNA. | 2,3 |
| 399 | CKS1B | Homo sapiens CDC28 protein kinase regulatory subunit 1B (CKS1B), mRNA. | 2,3 |
| 400 | CDKN3 | Homo sapiens cyclin-dependent kinase inhibitor 3 (CDK2-associated dual specificity phosphatase) (CDKN3), mRNA. | 2,3 |
| 401 | NFIA | Homo sapiens nuclear factor I/A (NFIA), mRNA. | 2,4 |
| 402 | MTUS1 | Homo sapiens mitochondrial tumor suppressor 1 (MTUS1), nuclear gene encoding mitochondrial protein, transcript variant 1, mRNA. | 2,4 |
| 403 | FAM38B | Homo sapiens family with sequence similarity 38, member B (FAM38B), mRNA. | 2,3 |
| 404 | CTSH | Homo sapiens cathepsin H (CTSH), transcript variant 1, mRNA. | 2,4 |
| 405 | CUBN | Homo sapiens cubilin (intrinsic factor-cobalamin receptor) (CUBN), mRNA. | 2,4 |
| 406 | ACSL5 | Homo sapiens acyl-CoA synthetase long-chain family member 5 (ACSL5), transcript variant 1, mRNA. | 2,3 |
| 407 | IL17RB | Homo sapiens interleukin 17 receptor B (IL17RB), mRNA. | 2,4 |
| 408 | CACYBP | Homo sapiens calcyclin binding protein (CACYBP), transcript variant 1, mRNA. | 2,3 |
| 409 | CDH13 | Homo sapiens cadherin 13, H-cadherin (heart) (CDH13), mRNA. | 2,3 |
| 410 | DIO2 | Homo sapiens deiodinase, iodothyronine, type II (DIO2), transcript variant 3, mRNA. | 2,6 |
| 411 | FUT4 | Homo sapiens fucosyltransferase 4 (alpha (1,3) fucosyltransferase, myeloid-specific) (FUT4), mRNA. | 2,3 |
| 412 | CNPY4 | Homo sapiens canopy 4 homolog (zebrafish) (CNPY4), mRNA. | 2,3 |
| 413 | NDRG2 | Homo sapiens NDRG family member 2 (NDRG2), transcript variant 6, mRNA. | 2,4 |
| 414 | C17orf97 | Homo sapiens chromosome 17 open reading frame 97 (C17orf97), mRNA. | 2,3 |
| 415 | MCM4 | Homo sapiens minichromosome maintenance complex component 4 (MCM4), transcript variant 1, mRNA. | 2,2 |
| 416 | FOXD2 | Homo sapiens forkhead box D2 (FOXD2), mRNA. | 2,3 |
| 417 | MAT2B | Homo sapiens methionine adenosyltransferase II, beta (MAT2B), transcript variant 1, mRNA. | 2,3 |
| 418 | ADAMTS1 | Homo sapiens ADAM metallopeptidase with thrombospondin type 1 motif, 1 (ADAMTS1), mRNA. | 2,3 |
| 419 | TIPARP | Homo sapiens TCDD-inducible poly(ADP-ribose) polymerase (TIPARP), mRNA. | 2,6 |
| 420 | WFDC1 | Homo sapiens WAP four-disulfide core domain 1 (WFDC1), mRNA. | 2,7 |
| 421 | CPNE8 | Homo sapiens copine VIII (CPNE8), mRNA. | 2,3 |
| 422 | SULF1 | Homo sapiens sulfatase 1 (SULF1), mRNA. | 2,6 |
| 423 | LRRC20 | Homo sapiens leucine rich repeat containing 20 (LRRC20), transcript variant 2, mRNA. | 2,2 |
| 424 | ZNF33B | Homo sapiens zinc finger protein 33B (ZNF33B), mRNA. | 2,2 |
| 425 | PQLC3 | Homo sapiens PQ loop repeat containing 3 (PQLC3), mRNA. | 2,2 |
| 426 | HMMR | Homo sapiens hyaluronan-mediated motility receptor (RHAMM) (HMMR), transcript variant 1, mRNA. | 2,3 |
| 427 | LOC654053 | PREDICTED: Homo sapiens similar to hypothetical LOC389634 (LOC654053), mRNA. | 2,6 |
