# Supplementary material for: Similar Properties of Chondrocytes from Osteoarthritis Joints and Mesenchymal Stem Cells from Healthy Donors for Tissue Engineering of Articular Cartilage
Source: PLoS One. 2013 May 9;8(5):e62994. doi: 10.1371/journal.pone.0062994 (PMC3650033; doi:10.1371/journal.pone.0062994)
Supplement: Table S2 — Genes upregulated in human bone marrow (BM)-MSCs embedded in alginate scaffold with chondrogenic medium after 21 days. (DOC) [file pone.0062994.s002.doc]

**Supplementary Table S2.** Genes upregulated in human bone marrow-MSCs embedded in alginate scaffold with chondrogenic medium after 21 days

| **Rank** | **Symbol** | **DEFINITION** | **Fold change** |
| --- | --- | --- | --- |
| 1 | PTGES | Homo sapiens prostaglandin E synthase (PTGES), mRNA. | 29,1 |
| 2 | ODZ3 | Homo sapiens odz, odd Oz/ten-m homolog 3 (Drosophila) (ODZ3), mRNA. | 17,5 |
| 3 | S100P | Homo sapiens S100 calcium binding protein P (S100P), mRNA. | 15,4 |
| 4 | PAMR1 | Homo sapiens peptidase domain containing associated with muscle regeneration 1 (PAMR1), transcript variant 1, mRNA. | 14,1 |
| 5 | CDH15 | Homo sapiens cadherin 15, type 1, M-cadherin (myotubule) (CDH15), mRNA. | 13,5 |
| 6 | IGFBP5 | Homo sapiens insulin-like growth factor binding protein 5 (IGFBP5), mRNA. | 14,6 |
| 7 | CA9 | Homo sapiens carbonic anhydrase IX (CA9), mRNA. | 13,5 |
| 8 | EEF1A2 | Homo sapiens eukaryotic translation elongation factor 1 alpha 2 (EEF1A2), mRNA. | 13,6 |
| 9 | CXCL14 | Homo sapiens chemokine (C-X-C motif) ligand 14 (CXCL14), mRNA. | 14,0 |
| 10 | SLC13A5 | Homo sapiens solute carrier family 13 (sodium-dependent citrate transporter), member 5 (SLC13A5), mRNA. | 10,8 |
| 11 | IGFBP5 | Homo sapiens insulin-like growth factor binding protein 5 (IGFBP5), mRNA. | 12,8 |
| 12 | CA12 | Homo sapiens carbonic anhydrase XII (CA12), transcript variant 1, mRNA. | 10,3 |
| 13 | HOXB7 | Homo sapiens homeobox B7 (HOXB7), mRNA. | 9,8 |
| 14 | CA12 | Homo sapiens carbonic anhydrase XII (CA12), transcript variant 1, mRNA. | 9,5 |
| 15 | SLAMF9 | Homo sapiens SLAM family member 9 (SLAMF9), mRNA. | 9,1 |
| 16 | CTSD | Homo sapiens cathepsin D (CTSD), mRNA. | 8,4 |
| 17 | MATN4 | Homo sapiens matrilin 4 (MATN4), transcript variant 3, mRNA. | 8,7 |
| 18 | IBSP | Homo sapiens integrin-binding sialoprotein (bone sialoprotein, bone sialoprotein II) (IBSP), mRNA. | 9,6 |
| 19 | ENO2 | Homo sapiens enolase 2 (gamma, neuronal) (ENO2), mRNA. | 8,2 |
| 20 | LOC646345 | PREDICTED: Homo sapiens hypothetical protein LOC646345 (LOC646345), mRNA. | 7,9 |
| 21 | RUNX3 | Homo sapiens runt-related transcription factor 3 (RUNX3), transcript variant 2, mRNA. | 7,7 |
| 22 | LOC650494 | PREDICTED: Homo sapiens hypothetical protein LOC650494 (LOC650494), mRNA. | 7,7 |
| 23 | HOXB2 | Homo sapiens homeobox B2 (HOXB2), mRNA. | 7,1 |
| 24 | SPP1 | Homo sapiens secreted phosphoprotein 1 (SPP1), transcript variant 1, mRNA. | 10,2 |
| 25 | TUBB3 | Homo sapiens tubulin, beta 3 (TUBB3), mRNA. | 7,3 |
| 26 | IRX5 | Homo sapiens iroquois homeobox 5 (IRX5), mRNA. | 6,8 |
| 27 | FLJ39632 | PREDICTED: Homo sapiens misc_RNA (FLJ39632), miscRNA. | 7,0 |
| 28 | DPYSL4 | Homo sapiens dihydropyrimidinase-like 4 (DPYSL4), mRNA. | 7,1 |
| 29 | GDF15 | Homo sapiens growth differentiation factor 15 (GDF15), mRNA. | 6,6 |
| 30 | CDH2 | Homo sapiens cadherin 2, type 1, N-cadherin (neuronal) (CDH2), mRNA. | 6,9 |
| 31 | COL10A1 | Homo sapiens collagen, type X, alpha 1 (COL10A1), mRNA. | 6,6 |
| 32 | SPP1 | Homo sapiens secreted phosphoprotein 1 (SPP1), transcript variant 2, mRNA. | 9,1 |
| 33 | TGM2 | Homo sapiens transglutaminase 2 (C polypeptide, protein-glutamine-gamma-glutamyltransferase) (TGM2), transcript variant 1, mRNA. | 6,4 |
| 34 | G0S2 | Homo sapiens G0/G1switch 2 (G0S2), mRNA. | 6,2 |
| 35 | PIM1 | Homo sapiens pim-1 oncogene (PIM1), mRNA. | 6,1 |
| 36 | GPC3 | Homo sapiens glypican 3 (GPC3), mRNA. | 5,8 |
| 37 | ANKRD33 | Homo sapiens ankyrin repeat domain 33 (ANKRD33), mRNA. | 6,0 |
| 38 | PANX3 | Homo sapiens pannexin 3 (PANX3), mRNA. | 8,0 |
| 39 | MATN4 | Homo sapiens matrilin 4 (MATN4), transcript variant 3, mRNA. | 5,9 |
| 40 | AOC2 | Homo sapiens amine oxidase, copper containing 2 (retina-specific) (AOC2), transcript variant 1, mRNA. | 5,9 |
| 41 | ALPL | Homo sapiens alkaline phosphatase, liver/bone/kidney (ALPL), transcript variant 1, mRNA. | 6,1 |
| 42 | IRF6 | Homo sapiens interferon regulatory factor 6 (IRF6), mRNA. | 5,6 |
| 43 | PPA1 | Homo sapiens pyrophosphatase (inorganic) 1 (PPA1), mRNA. | 5,7 |
| 44 | PGF | Homo sapiens placental growth factor (PGF), mRNA. | 5,6 |
| 45 | GAPDH | Homo sapiens glyceraldehyde-3-phosphate dehydrogenase (GAPDH), mRNA. | 5,6 |
| 46 | TMEM119 | Homo sapiens transmembrane protein 119 (TMEM119), mRNA. | 6,2 |
| 47 | SERPINE2 | Homo sapiens serpin peptidase inhibitor, clade E (nexin, plasminogen activator inhibitor type 1), member 2 (SERPINE2), mRNA. | 5,6 |
| 48 | TRPV4 | Homo sapiens transient receptor potential cation channel, subfamily V, member 4 (TRPV4), transcript variant 2, mRNA. | 5,4 |
| 49 | PITPNC1 | Homo sapiens phosphatidylinositol transfer protein, cytoplasmic 1 (PITPNC1), transcript variant 2, mRNA. | 5,4 |
| 50 | A2M | Homo sapiens alpha-2-macroglobulin (A2M), mRNA. | 5,6 |
| 51 | SP7 | Homo sapiens Sp7 transcription factor (SP7), mRNA. | 5,1 |
| 52 | RCOR2 | Homo sapiens REST corepressor 2 (RCOR2), mRNA. | 5,1 |
| 53 | SMOX | Homo sapiens spermine oxidase (SMOX), transcript variant 2, mRNA. | 5,1 |
| 54 | KRT16 | Homo sapiens keratin 16 (focal non-epidermolytic palmoplantar keratoderma) (KRT16), mRNA. | 5,0 |
| 55 | TGFBI | Homo sapiens transforming growth factor, beta-induced, 68kDa (TGFBI), mRNA. | 5,1 |
| 56 | HLA-A | Homo sapiens major histocompatibility complex, class I, A (HLA-A), mRNA. | 4,8 |
| 57 | MSX1 | Homo sapiens msh homeobox 1 (MSX1), mRNA. | 5,0 |
| 58 | WNT4 | Homo sapiens wingless-type MMTV integration site family, member 4 (WNT4), mRNA. | 4,9 |
| 59 | OSR1 | Homo sapiens odd-skipped related 1 (Drosophila) (OSR1), mRNA. | 5,0 |
| 60 | NPAS1 | Homo sapiens neuronal PAS domain protein 1 (NPAS1), mRNA. | 5,0 |
| 61 | PAMR1 | Homo sapiens peptidase domain containing associated with muscle regeneration 1 (PAMR1), transcript variant 1, mRNA. | 4,6 |
| 62 | TYRO3 | Homo sapiens TYRO3 protein tyrosine kinase (TYRO3), mRNA. | 4,6 |
| 63 | PANX2 | Homo sapiens pannexin 2 (PANX2), mRNA. | 4,5 |
| 64 | WNT5B | Homo sapiens wingless-type MMTV integration site family, member 5B (WNT5B), transcript variant 1, mRNA. | 4,5 |
| 65 | TSPAN13 | Homo sapiens tetraspanin 13 (TSPAN13), mRNA. | 4,9 |
| 66 | SERPINH1 | Homo sapiens serpin peptidase inhibitor, clade H (heat shock protein 47), member 1, (collagen binding protein 1) (SERPINH1), mRNA. | 4,5 |
| 67 | PFKP | Homo sapiens phosphofructokinase, platelet (PFKP), mRNA. | 4,5 |
| 68 | VEGFA | Homo sapiens vascular endothelial growth factor A (VEGFA), transcript variant 2, mRNA. | 4,4 |
| 69 | IRX5 | Homo sapiens iroquois homeobox protein 5 (IRX5), mRNA. | 4,4 |
| 70 | LOC400879 | PREDICTED: Homo sapiens hypothetical LOC400879, transcript variant 2 (LOC400879), mRNA. | 4,5 |
| 71 | EYA2 | Homo sapiens eyes absent homolog 2 (Drosophila) (EYA2), transcript variant 4, mRNA. | 4,2 |
| 72 | CDKN1C | Homo sapiens cyclin-dependent kinase inhibitor 1C (p57, Kip2) (CDKN1C), mRNA. | 4,3 |
| 73 | SLC7A5 | Homo sapiens solute carrier family 7 (cationic amino acid transporter, y+ system), member 5 (SLC7A5), mRNA. | 4,2 |
| 74 | LOC642477 | PREDICTED: Homo sapiens hypothetical protein LOC642477, transcript variant 2 (LOC642477), mRNA. | 4,3 |
| 75 | WDR86 | Homo sapiens WD repeat domain 86 (WDR86), mRNA. | 4,2 |
| 76 | CITED4 | Homo sapiens Cbp/p300-interacting transactivator, with Glu/Asp-rich carboxy-terminal domain, 4 (CITED4), mRNA. | 4,2 |
| 77 | LEF1 | Homo sapiens lymphoid enhancer-binding factor 1 (LEF1), mRNA. | 4,4 |
| 78 | IRX3 | Homo sapiens iroquois homeobox 3 (IRX3), mRNA. | 4,6 |
| 79 | RASD1 | Homo sapiens RAS, dexamethasone-induced 1 (RASD1), mRNA. | 4,2 |
| 80 | IHH | Homo sapiens Indian hedgehog homolog (Drosophila) (IHH), mRNA. | 4,2 |
| 81 | CD24 | Homo sapiens CD24 molecule (CD24), mRNA. | 4,6 |
| 82 | LAMA4 | Homo sapiens laminin, alpha 4 (LAMA4), mRNA. | 4,2 |
| 83 | LGALS3BP | Homo sapiens lectin, galactoside-binding, soluble, 3 binding protein (LGALS3BP), mRNA. | 4,0 |
| 84 | NES | Homo sapiens nestin (NES), mRNA. | 4,2 |
| 85 | DCLK1 | Homo sapiens doublecortin-like kinase 1 (DCLK1), mRNA. | 4,4 |
| 86 | WNT11 | Homo sapiens wingless-type MMTV integration site family, member 11 (WNT11), mRNA. | 3,9 |
| 87 | PMP2 | Homo sapiens peripheral myelin protein 2 (PMP2), mRNA. | 4,1 |
| 88 | CACNA1H | Homo sapiens calcium channel, voltage-dependent, T type, alpha 1H subunit (CACNA1H), transcript variant 1, mRNA. | 3,8 |
| 89 | SLC27A1 | Homo sapiens solute carrier family 27 (fatty acid transporter), member 1 (SLC27A1), mRNA. | 3,9 |
| 90 | GAPDH | Homo sapiens glyceraldehyde-3-phosphate dehydrogenase (GAPDH), mRNA. | 3,9 |
| 91 | LOC440160 | PREDICTED: Homo sapiens hypothetical LOC440160 (LOC440160), mRNA. | 3,9 |
| 92 | LEF1 | Homo sapiens lymphoid enhancer-binding factor 1 (LEF1), mRNA. | 4,1 |
| 93 | FZD9 | Homo sapiens frizzled homolog 9 (Drosophila) (FZD9), mRNA. | 3,8 |
| 94 | CLDN11 | Homo sapiens claudin 11 (oligodendrocyte transmembrane protein) (CLDN11), mRNA. | 4,0 |
| 95 | SCUBE3 | Homo sapiens signal peptide, CUB domain, EGF-like 3 (SCUBE3), mRNA. | 3,9 |
| 96 | SRPX | Homo sapiens sushi-repeat-containing protein, X-linked (SRPX), mRNA. | 3,9 |
| 97 | RAC3 | Homo sapiens ras-related C3 botulinum toxin substrate 3 (rho family, small GTP binding protein Rac3) (RAC3), mRNA. | 3,7 |
| 98 | APCDD1L | Homo sapiens adenomatosis polyposis coli down-regulated 1-like (APCDD1L), mRNA. | 4,4 |
| 99 | LOX | Homo sapiens lysyl oxidase (LOX), mRNA. | 3,8 |
| 100 | MXRA7 | Homo sapiens matrix-remodelling associated 7 (MXRA7), transcript variant 2, mRNA. | 3,8 |
| 101 | KDELR2 | Homo sapiens KDEL (Lys-Asp-Glu-Leu) endoplasmic reticulum protein retention receptor 2 (KDELR2), transcript variant 1, mRNA. | 3,7 |
| 102 | HLA-A | Homo sapiens major histocompatibility complex, class I, A (HLA-A), mRNA. | 3,6 |
| 103 | MAFB | Homo sapiens v-maf musculoaponeurotic fibrosarcoma oncogene homolog B (avian) (MAFB), mRNA. | 3,8 |
| 104 | TF | Homo sapiens transferrin (TF), mRNA. | 3,6 |
| 105 | BMP2 | Homo sapiens bone morphogenetic protein 2 (BMP2), mRNA. | 3,6 |
| 106 | EBF3 | Homo sapiens early B-cell factor 3 (EBF3), mRNA. | 3,6 |
| 107 | AOC2 | Homo sapiens amine oxidase, copper containing 2 (retina-specific) (AOC2), transcript variant 2, mRNA. | 3,7 |
| 108 | FGF11 | Homo sapiens fibroblast growth factor 11 (FGF11), mRNA. | 3,6 |
| 109 | PPARG | Homo sapiens peroxisome proliferator-activated receptor gamma (PPARG), transcript variant 2, mRNA. | 3,6 |
| 110 | SCARB1 | Homo sapiens scavenger receptor class B, member 1 (SCARB1), mRNA. | 3,6 |
| 111 | HLA-H | Homo sapiens major histocompatibility complex, class I, H (pseudogene) (HLA-H), non-coding RNA. | 3,6 |
| 112 | SEL1L3 | Homo sapiens sel-1 suppressor of lin-12-like 3 (C. elegans) (SEL1L3), mRNA. | 3,6 |
| 113 | CFD | Homo sapiens complement factor D (adipsin) (CFD), mRNA. | 3,8 |
| 114 | THBS2 | Homo sapiens thrombospondin 2 (THBS2), mRNA. | 3,7 |
| 115 | KCNG1 | Homo sapiens potassium voltage-gated channel, subfamily G, member 1 (KCNG1), mRNA. | 3,6 |
| 116 | GALNT5 | Homo sapiens UDP-N-acetyl-alpha-D-galactosamine:polypeptide N-acetylgalactosaminyltransferase 5 (GalNAc-T5) (GALNT5), mRNA. | 3,6 |
| 117 | RNF150 | Homo sapiens ring finger protein 150 (RNF150), mRNA. | 3,5 |
| 118 | FER1L4 | Homo sapiens fer-1-like 4 (C. elegans) (FER1L4) on chromosome 20. | 3,6 |
| 119 | LOC400578 | PREDICTED: Homo sapiens similar to Keratin, type I cytoskeletal 14 (Cytokeratin-14) (CK-14) (Keratin-14) (K14) (LOC400578), mRNA. | 3,5 |
| 120 | DNAJC12 | Homo sapiens DnaJ (Hsp40) homolog, subfamily C, member 12 (DNAJC12), transcript variant 1, mRNA. | 3,5 |
| 121 | SKAP2 | Homo sapiens src kinase associated phosphoprotein 2 (SKAP2), mRNA. | 3,5 |
| 122 | DKK1 | Homo sapiens dickkopf homolog 1 (Xenopus laevis) (DKK1), mRNA. | 3,7 |
| 123 | TSPAN13 | Homo sapiens tetraspanin 13 (TSPAN13), mRNA. | 3,5 |
| 124 | IGFBP4 | Homo sapiens insulin-like growth factor binding protein 4 (IGFBP4), mRNA. | 4,0 |
| 125 | AOC2 | Homo sapiens amine oxidase, copper containing 2 (retina-specific) (AOC2), transcript variant 1, mRNA. | 3,5 |
| 126 | PTGDS | Homo sapiens prostaglandin D2 synthase 21kDa (brain) (PTGDS), mRNA. | 3,4 |
| 127 | CD320 | Homo sapiens CD320 molecule (CD320), mRNA. | 3,5 |
| 128 | TRIM22 | Homo sapiens tripartite motif-containing 22 (TRIM22), mRNA. | 3,4 |
| 129 | LOC440928 | PREDICTED: Homo sapiens hypothetical LOC440928 (LOC440928), mRNA. | 3,4 |
| 130 | MATN4 | Homo sapiens matrilin 4 (MATN4), transcript variant 1, mRNA. | 3,3 |
| 131 | DRD4 | Homo sapiens dopamine receptor D4 (DRD4), mRNA. | 3,5 |
| 132 | LDB2 | Homo sapiens LIM domain binding 2 (LDB2), mRNA. | 3,4 |
| 133 | ZNF503 | Homo sapiens zinc finger protein 503 (ZNF503), mRNA. | 3,4 |
| 134 | TRPV4 | Homo sapiens transient receptor potential cation channel, subfamily V, member 4 (TRPV4), transcript variant 1, mRNA. | 3,3 |
| 135 | RAC2 | Homo sapiens ras-related C3 botulinum toxin substrate 2 (rho family, small GTP binding protein Rac2) (RAC2), mRNA. | 3,4 |
| 136 | P4HA1 | Homo sapiens procollagen-proline, 2-oxoglutarate 4-dioxygenase (proline 4-hydroxylase), alpha polypeptide I (P4HA1), transcript variant 1, mRNA. | 3,3 |
| 137 | TXNDC5 | Homo sapiens thioredoxin domain containing 5 (TXNDC5), transcript variant 2, mRNA. | 3,3 |
| 138 | GPC1 | Homo sapiens glypican 1 (GPC1), mRNA. | 3,3 |
| 139 | TMEM145 | Homo sapiens transmembrane protein 145 (TMEM145), mRNA. | 3,4 |
| 140 | SMOX | Homo sapiens spermine oxidase (SMOX), transcript variant 4, mRNA. | 3,4 |
| 141 | ZFHX4 | Homo sapiens zinc finger homeobox 4 (ZFHX4), mRNA. | 3,6 |
| 142 | VARS | Homo sapiens valyl-tRNA synthetase (VARS), nuclear gene encoding mitochondrial protein, mRNA. | 3,3 |
| 143 | AK3L1 | Homo sapiens adenylate kinase 3-like 1 (AK3L1), nuclear gene encoding mitochondrial protein, transcript variant 6, mRNA. | 3,3 |
| 144 | MAP2K1 | Homo sapiens mitogen-activated protein kinase kinase 1 (MAP2K1), mRNA. | 3,3 |
| 145 | PTH1R | Homo sapiens parathyroid hormone 1 receptor (PTH1R), mRNA. | 3,3 |
| 146 | LOC643008 | PREDICTED: Homo sapiens PP12104, transcript variant 1 (LOC643008), mRNA. | 3,3 |
| 147 |  | Homo sapiens cDNA clone IMAGE:4837650 | 3,2 |
| 148 | CSRP2 | Homo sapiens cysteine and glycine-rich protein 2 (CSRP2), mRNA. | 3,6 |
| 149 | LOC642282 | PREDICTED: Homo sapiens similar to Tektin-3, transcript variant 1 (LOC642282), mRNA. | 3,4 |
| 150 | FLNC | Homo sapiens filamin C, gamma (actin binding protein 280) (FLNC), mRNA. | 3,2 |
| 151 | CART1 | Homo sapiens cartilage paired-class homeoprotein 1 (CART1), mRNA. | 3,1 |
| 152 | DBNDD1 | Homo sapiens dysbindin (dystrobrevin binding protein 1) domain containing 1 (DBNDD1), transcript variant 1, mRNA. | 3,9 |
| 153 | LOC728715 | PREDICTED: Homo sapiens similar to hCG38149 (LOC728715), mRNA. | 3,2 |
| 154 | IGF2BP2 | Homo sapiens insulin-like growth factor 2 mRNA binding protein 2 (IGF2BP2), transcript variant 1, mRNA. | 3,1 |
| 155 | ATF3 | Homo sapiens activating transcription factor 3 (ATF3), transcript variant 4, mRNA. | 3,2 |
| 156 | TRIB3 | Homo sapiens tribbles homolog 3 (Drosophila) (TRIB3), mRNA. | 3,6 |
| 157 | DKFZp451A211 | Homo sapiens DKFZp451A211 protein (DKFZp451A211), mRNA. | 3,2 |
| 158 | TNFRSF12A | Homo sapiens tumor necrosis factor receptor superfamily, member 12A (TNFRSF12A), mRNA. | 3,8 |
| 159 | CYGB | Homo sapiens cytoglobin (CYGB), mRNA. | 3,2 |
| 160 | SPAG4 | Homo sapiens sperm associated antigen 4 (SPAG4), mRNA. | 3,1 |
| 161 | LGR6 | Homo sapiens leucine-rich repeat-containing G protein-coupled receptor 6 (LGR6), transcript variant 1, mRNA. | 3,1 |
| 162 | GPI | Homo sapiens glucose phosphate isomerase (GPI), mRNA. | 3,1 |
| 163 | CBX4 | Homo sapiens chromobox homolog 4 (Pc class homolog, Drosophila) (CBX4), mRNA. | 3,1 |
| 164 | P4HA2 | Homo sapiens prolyl 4-hydroxylase, alpha polypeptide II (P4HA2), transcript variant 3, mRNA. | 3,1 |
| 165 | BNIP3 | Homo sapiens BCL2/adenovirus E1B 19kDa interacting protein 3 (BNIP3), nuclear gene encoding mitochondrial protein, mRNA. | 3,2 |
| 166 | TPI1 | Homo sapiens triosephosphate isomerase 1 (TPI1), mRNA. | 3,1 |
| 167 | AK3L1 | Homo sapiens adenylate kinase 3-like 1 (AK3L1), nuclear gene encoding mitochondrial protein, transcript variant 7, mRNA. | 3,1 |
| 168 | SH3BP4 | Homo sapiens SH3-domain binding protein 4 (SH3BP4), mRNA. | 3,1 |
| 169 | SCD | Homo sapiens stearoyl-CoA desaturase (delta-9-desaturase) (SCD), mRNA. | 3,1 |
| 170 | MGC102966 | PREDICTED: Homo sapiens similar to Keratin, type I cytoskeletal 16 (Cytokeratin-16) (CK-16) (Keratin-16) (K16) (MGC102966), misc RNA. | 2,9 |
| 171 | DNAJC12 | Homo sapiens DnaJ (Hsp40) homolog, subfamily C, member 12 (DNAJC12), transcript variant 2, mRNA. | 3,0 |
| 172 | PTPRF | Homo sapiens protein tyrosine phosphatase, receptor type, F (PTPRF), transcript variant 1, mRNA. | 3,1 |
| 173 | PLCD1 | Homo sapiens phospholipase C, delta 1 (PLCD1), mRNA. | 3,0 |
| 174 | FBLN1 | Homo sapiens fibulin 1 (FBLN1), transcript variant C, mRNA. | 3,0 |
| 175 | DCLK1 | Homo sapiens doublecortin-like kinase 1 (DCLK1), mRNA. | 3,2 |
| 176 | SLC8A3 | Homo sapiens solute carrier family 8 (sodium/calcium exchanger), member 3 (SLC8A3), transcript variant f, mRNA. | 3,6 |
| 177 | PITX2 | Homo sapiens paired-like homeodomain 2 (PITX2), transcript variant 1, mRNA. | 2,9 |
| 178 | IRX2 | Homo sapiens iroquois homeobox 2 (IRX2), mRNA. | 3,0 |
| 179 | DHRS11 | Homo sapiens dehydrogenase/reductase (SDR family) member 11 (DHRS11), mRNA. | 3,0 |
| 180 | LOXL4 | Homo sapiens lysyl oxidase-like 4 (LOXL4), mRNA. | 3,3 |
| 181 | HCG4 | Homo sapiens HLA complex group 4 (HCG4), non-coding RNA. | 3,0 |
| 182 | ENG | Homo sapiens endoglin (Osler-Rendu-Weber syndrome 1) (ENG), mRNA. | 3,0 |
| 183 | TPI1 | Homo sapiens triosephosphate isomerase 1 (TPI1), mRNA. | 2,9 |
| 184 | FGFR4 | Homo sapiens fibroblast growth factor receptor 4 (FGFR4), transcript variant 3, mRNA. | 3,0 |
| 185 | ADM | Homo sapiens adrenomedullin (ADM), mRNA. | 3,0 |
| 186 | HOXB3 | Homo sapiens homeobox B3 (HOXB3), mRNA. | 2,9 |
| 187 | SLIT3 | Homo sapiens slit homolog 3 (Drosophila) (SLIT3), mRNA. | 2,9 |
| 188 | FAM129A | Homo sapiens family with sequence similarity 129, member A (FAM129A), transcript variant 2, mRNA. | 3,0 |
| 189 | MICA | Homo sapiens MHC class I polypeptide-related sequence A (MICA), mRNA. | 2,9 |
| 190 | SEC14L2 | Homo sapiens SEC14-like 2 (S. cerevisiae) (SEC14L2), mRNA. | 2,9 |
| 191 | NFASC | Homo sapiens neurofascin homolog (chicken) (NFASC), transcript variant 5, mRNA. | 2,9 |
| 192 | BIN1 | Homo sapiens bridging integrator 1 (BIN1), transcript variant 1, mRNA. | 2,8 |
| 193 | VEGFA | Homo sapiens vascular endothelial growth factor A (VEGFA), transcript variant 3, mRNA. | 2,9 |
| 194 | KCNS1 | Homo sapiens potassium voltage-gated channel, delayed-rectifier, subfamily S, member 1 (KCNS1), mRNA. | 3,1 |
| 195 | PTPRD | Homo sapiens protein tyrosine phosphatase, receptor type, D (PTPRD), transcript variant 2, mRNA. | 2,9 |
| 196 | UNC5B | Homo sapiens unc-5 homolog B (C. elegans) (UNC5B), mRNA. | 2,8 |
| 197 | SLFN11 | Homo sapiens schlafen family member 11 (SLFN11), mRNA. | 3,0 |
| 198 | SHC4 | Homo sapiens SHC (Src homology 2 domain containing) family, member 4 (SHC4), mRNA. | 2,9 |
| 199 | LOC650369 | PREDICTED: Homo sapiens similar to family with sequence similarity 60, member A (LOC650369), mRNA. | 2,8 |
| 200 | FLRT3 | Homo sapiens fibronectin leucine rich transmembrane protein 3 (FLRT3), transcript variant 2, mRNA. | 2,8 |
| 201 | SERINC2 | Homo sapiens serine incorporator 2 (SERINC2), mRNA. | 2,9 |
| 202 | DBNDD1 | Homo sapiens dysbindin (dystrobrevin binding protein 1) domain containing 1 (DBNDD1), transcript variant 1, mRNA. | 3,3 |
| 203 | HLA-F | Homo sapiens major histocompatibility complex, class I, F (HLA-F), mRNA. | 2,9 |
| 204 | UCHL1 | Homo sapiens ubiquitin carboxyl-terminal esterase L1 (ubiquitin thiolesterase) (UCHL1), mRNA. | 2,9 |
| 205 | CSNK1E | Homo sapiens casein kinase 1, epsilon (CSNK1E), transcript variant 1, mRNA. | 2,8 |
| 206 | PLOD3 | Homo sapiens procollagen-lysine, 2-oxoglutarate 5-dioxygenase 3 (PLOD3), mRNA. | 2,8 |
| 207 | EXTL1 | Homo sapiens exostoses (multiple)-like 1 (EXTL1), mRNA. | 3,0 |
| 208 | CHST3 | Homo sapiens carbohydrate (chondroitin 6) sulfotransferase 3 (CHST3), mRNA. | 2,9 |
| 209 | CCND2 | Homo sapiens cyclin D2 (CCND2), mRNA. | 2,8 |
| 210 | SPARCL1 | Homo sapiens SPARC-like 1 (mast9, hevin) (SPARCL1), mRNA. | 3,3 |
| 211 | KIAA0090 | Homo sapiens KIAA0090 (KIAA0090), mRNA. | 2,8 |
| 212 | GALE | Homo sapiens UDP-galactose-4-epimerase (GALE), transcript variant 1, mRNA. | 2,8 |
| 213 | GBE1 | Homo sapiens glucan (1,4-alpha-), branching enzyme 1 (glycogen branching enzyme, Andersen disease, glycogen storage disease type IV) (GBE1), mRNA. | 2,9 |
| 214 | RRBP1 | Homo sapiens ribosome binding protein 1 homolog 180kDa (dog) (RRBP1), transcript variant 1, mRNA. | 2,8 |
| 215 | PFKFB4 | Homo sapiens 6-phosphofructo-2-kinase/fructose-2,6-biphosphatase 4 (PFKFB4), mRNA. | 3,0 |
| 216 | P4HA2 | Homo sapiens prolyl 4-hydroxylase, alpha polypeptide II (P4HA2), transcript variant 2, mRNA. | 2,8 |
| 217 | MIF | Homo sapiens macrophage migration inhibitory factor (glycosylation-inhibiting factor) (MIF), mRNA. | 2,8 |
| 218 | HYOU1 | Homo sapiens hypoxia up-regulated 1 (HYOU1), mRNA. | 2,7 |
| 219 | DNAJC12 | Homo sapiens DnaJ (Hsp40) homolog, subfamily C, member 12 (DNAJC12), transcript variant 1, mRNA. | 2,8 |
| 220 | LOC653344 | PREDICTED: Homo sapiens similar to cis-Golgi matrix protein GM130, transcript variant 2 (LOC653344), mRNA. | 2,7 |
| 221 | LOXL3 | Homo sapiens lysyl oxidase-like 3 (LOXL3), mRNA. | 2,8 |
| 222 | PAK2 | PREDICTED: Homo sapiens p21 (CDKN1A)-activated kinase 2 (PAK2), mRNA. | 2,7 |
| 223 | C1orf165 | Homo sapiens chromosome 1 open reading frame 165 (C1orf165), mRNA. | 2,9 |
| 224 | BIN1 | Homo sapiens bridging integrator 1 (BIN1), transcript variant 4, mRNA. | 2,7 |
| 225 | RASSF7 | Homo sapiens Ras association (RalGDS/AF-6) domain family (N-terminal) member 7 (RASSF7), mRNA. | 2,7 |
| 226 | DHCR7 | Homo sapiens 7-dehydrocholesterol reductase (DHCR7), mRNA. | 2,8 |
| 227 | UNC5B | Homo sapiens unc-5 homolog B (C. elegans) (UNC5B), mRNA. | 2,7 |
| 228 | FAM162A | Homo sapiens family with sequence similarity 162, member A (FAM162A), mRNA. | 2,7 |
| 229 | INSC | Homo sapiens inscuteable homolog (Drosophila) (INSC), transcript variant 2, mRNA. | 3,0 |
| 230 | BOK | Homo sapiens BCL2-related ovarian killer (BOK), mRNA. | 2,7 |
| 231 | F12 | Homo sapiens coagulation factor XII (Hageman factor) (F12), mRNA. | 2,7 |
| 232 | ALDOA | Homo sapiens aldolase A, fructose-bisphosphate (ALDOA), transcript variant 3, mRNA. | 2,7 |
| 233 | HLA-G | Homo sapiens HLA-G histocompatibility antigen, class I, G (HLA-G), mRNA. | 2,7 |
| 234 | CRELD1 | Homo sapiens cysteine-rich with EGF-like domains 1 (CRELD1), transcript variant 1, mRNA. | 2,8 |
| 235 | P4HA2 | Homo sapiens prolyl 4-hydroxylase, alpha polypeptide II (P4HA2), transcript variant 3, mRNA. | 2,7 |
| 236 | COL27A1 | Homo sapiens collagen, type XXVII, alpha 1 (COL27A1), mRNA. | 2,6 |
| 237 | CLEC3A | Homo sapiens C-type lectin domain family 3, member A (CLEC3A), mRNA. | 3,7 |
| 238 | FHL2 | Homo sapiens four and a half LIM domains 2 (FHL2), transcript variant 4, mRNA. | 2,6 |
| 239 | PHOSPHO1 | Homo sapiens phosphatase, orphan 1 (PHOSPHO1), mRNA. | 2,8 |
| 240 | TUBA4A | Homo sapiens tubulin, alpha 4a (TUBA4A), mRNA. | 2,7 |
| 241 | HOMER2 | Homo sapiens homer homolog 2 (Drosophila) (HOMER2), transcript variant 3, mRNA. | 2,7 |
| 242 | FN1 | Homo sapiens fibronectin 1 (FN1), transcript variant 6, mRNA. | 2,6 |
| 243 | KAZALD1 | Homo sapiens Kazal-type serine peptidase inhibitor domain 1 (KAZALD1), mRNA. | 2,8 |
| 244 | TNFRSF10B | Homo sapiens tumor necrosis factor receptor superfamily, member 10b (TNFRSF10B), transcript variant 1, mRNA. | 2,6 |
| 245 | MARCKSL1 | Homo sapiens MARCKS-like 1 (MARCKSL1), mRNA. | 2,7 |
| 246 | PPP1R14B | Homo sapiens protein phosphatase 1, regulatory (inhibitor) subunit 14B (PPP1R14B), mRNA. | 2,6 |
| 247 | SPARCL1 | Homo sapiens SPARC-like 1 (mast9, hevin) (SPARCL1), mRNA. | 3,1 |
| 248 | ERO1L | Homo sapiens ERO1-like (S. cerevisiae) (ERO1L), mRNA. | 2,7 |
| 249 | HYOU1 | Homo sapiens hypoxia up-regulated 1 (HYOU1), mRNA. | 2,6 |
| 250 | FSTL3 | Homo sapiens follistatin-like 3 (secreted glycoprotein) (FSTL3), mRNA. | 2,6 |
| 251 | TNFRSF19 | Homo sapiens tumor necrosis factor receptor superfamily, member 19 (TNFRSF19), transcript variant 2, mRNA. | 3,1 |
| 252 | FBLN1 | Homo sapiens fibulin 1 (FBLN1), transcript variant A, mRNA. | 2,6 |
| 253 | P4HB | Homo sapiens prolyl 4-hydroxylase, beta polypeptide (P4HB), mRNA. | 2,6 |
| 254 | SFRP1 | Homo sapiens secreted frizzled-related protein 1 (SFRP1), mRNA. | 3,1 |
| 255 | FN1 | Homo sapiens fibronectin 1 (FN1), transcript variant 3, mRNA. | 2,6 |
| 256 | TNFRSF21 | Homo sapiens tumor necrosis factor receptor superfamily, member 21 (TNFRSF21), mRNA. | 2,7 |
| 257 | INSC | Homo sapiens inscuteable homolog (Drosophila) (INSC), transcript variant 2, mRNA. | 2,7 |
| 258 | RASIP1 | Homo sapiens Ras interacting protein 1 (RASIP1), mRNA. | 2,6 |
| 259 |  | UI-H-BI1-adn-f-06-0-UI.s1 NCI_CGAP_Sub3 Homo sapiens cDNA clone IMAGE:2717339 3, mRNA sequence | 2,5 |
| 260 | TXNDC5 | Homo sapiens thioredoxin domain containing 5 (endoplasmic reticulum) (TXNDC5), transcript variant 1, mRNA. | 2,6 |
| 261 | CARS | Homo sapiens cysteinyl-tRNA synthetase (CARS), transcript variant 4, mRNA. | 2,6 |
| 262 | C20orf100 | Homo sapiens chromosome 20 open reading frame 100 (C20orf100), mRNA. | 3,0 |
| 263 | COL4A3BP | Homo sapiens collagen, type IV, alpha 3 (Goodpasture antigen) binding protein (COL4A3BP), transcript variant 1, mRNA. | 2,5 |
| 264 | HOXB5 | Homo sapiens homeobox B5 (HOXB5), mRNA. | 2,5 |
| 265 | CCND2 | Homo sapiens cyclin D2 (CCND2), mRNA. | 2,6 |
| 266 | AKT1 | Homo sapiens v-akt murine thymoma viral oncogene homolog 1 (AKT1), transcript variant 3, mRNA. | 2,6 |
| 267 | PGAM1 | Homo sapiens phosphoglycerate mutase 1 (brain) (PGAM1), mRNA. | 2,6 |
| 268 | TNK2 | Homo sapiens tyrosine kinase, non-receptor, 2 (TNK2), transcript variant 1, mRNA. | 2,6 |
| 269 | LONP1 | Homo sapiens lon peptidase 1, mitochondrial (LONP1), nuclear gene encoding mitochondrial protein, mRNA. | 2,5 |
| 270 | MKNK2 | Homo sapiens MAP kinase interacting serine/threonine kinase 2 (MKNK2), transcript variant 1, mRNA. | 2,5 |
| 271 | LOC732165 | PREDICTED: Homo sapiens similar to Triosephosphate isomerase (TIM) (Triose-phosphate isomerase), transcript variant 2 (LOC732165), mRNA. | 2,6 |
| 272 | ST3GAL5 | Homo sapiens ST3 beta-galactoside alpha-2,3-sialyltransferase 5 (ST3GAL5), transcript variant 2, mRNA. | 2,5 |
| 273 | PLOD1 | Homo sapiens procollagen-lysine 1, 2-oxoglutarate 5-dioxygenase 1 (PLOD1), mRNA. | 2,6 |
| 274 | MDK | Homo sapiens midkine (neurite growth-promoting factor 2) (MDK), transcript variant 1, mRNA. | 2,5 |
| 275 | GSK3B | Homo sapiens glycogen synthase kinase 3 beta (GSK3B), mRNA. | 2,5 |
| 276 | GAPDH | Homo sapiens glyceraldehyde-3-phosphate dehydrogenase (GAPDH), mRNA. | 2,5 |
| 277 | SGMS2 | Homo sapiens sphingomyelin synthase 2 (SGMS2), mRNA. | 2,8 |
| 278 | ACTN4 | Homo sapiens actinin, alpha 4 (ACTN4), mRNA. | 2,6 |
| 279 | LOC646786 | PREDICTED: Homo sapiens similar to Afadin (AF-6 protein) (LOC646786), mRNA. | 2,5 |
| 280 | C4orf32 | Homo sapiens chromosome 4 open reading frame 32 (C4orf32), mRNA. | 2,5 |
| 281 | PAWR | Homo sapiens PRKC, apoptosis, WT1, regulator (PAWR), mRNA. | 2,5 |
| 282 | SNAP25 | Homo sapiens synaptosomal-associated protein, 25kDa (SNAP25), transcript variant 2, mRNA. | 2,5 |
| 283 | SLC5A3 | Homo sapiens solute carrier family 5 (sodium/myo-inositol cotransporter), member 3 (SLC5A3), mRNA. | 2,6 |
| 284 | KCNH2 | Homo sapiens potassium voltage-gated channel, subfamily H (eag-related), member 2 (KCNH2), transcript variant 2, mRNA. | 2,5 |
| 285 | RASL11B | Homo sapiens RAS-like, family 11, member B (RASL11B), mRNA. | 2,8 |
| 286 | IFITM5 | Homo sapiens interferon induced transmembrane protein 5 (IFITM5), mRNA. | 3,0 |
| 287 | CKAP4 | Homo sapiens cytoskeleton-associated protein 4 (CKAP4), mRNA. | 2,6 |
| 288 | PCSK5 | Homo sapiens proprotein convertase subtilisin/kexin type 5 (PCSK5), mRNA. | 2,5 |
| 289 | PFKL | Homo sapiens phosphofructokinase, liver (PFKL), transcript variant 2, mRNA. | 2,5 |
| 290 | EXOSC5 | Homo sapiens exosome component 5 (EXOSC5), mRNA. | 2,5 |
| 291 | CUEDC1 | Homo sapiens CUE domain containing 1 (CUEDC1), mRNA. | 2,5 |
| 292 | ANKRD38 | Homo sapiens ankyrin repeat domain 38 (ANKRD38), mRNA. | 2,6 |
| 293 | PNMA2 | Homo sapiens paraneoplastic antigen MA2 (PNMA2), mRNA. | 2,7 |
| 294 | IRF2BP2 | Homo sapiens interferon regulatory factor 2 binding protein 2 (IRF2BP2), transcript variant 1, mRNA. | 2,5 |
| 295 | ADAM15 | Homo sapiens ADAM metallopeptidase domain 15 (ADAM15), transcript variant 4, mRNA. | 2,4 |
| 296 | VDAC1 | Homo sapiens voltage-dependent anion channel 1 (VDAC1), mRNA. | 2,4 |
| 297 | FZD2 | Homo sapiens frizzled homolog 2 (Drosophila) (FZD2), mRNA. | 2,5 |
| 298 | ETV5 | Homo sapiens ets variant gene 5 (ets-related molecule) (ETV5), mRNA. | 2,5 |
| 299 | GPR175 | Homo sapiens G protein-coupled receptor 175 (GPR175), mRNA. | 2,4 |
| 300 | LOC643031 | PREDICTED: Homo sapiens similar to NADH dehydrogenase subunit 5 (LOC643031), mRNA. | 2,4 |
| 301 | LOC644774 | PREDICTED: Homo sapiens similar to Phosphoglycerate kinase 1 (LOC644774), mRNA. | 2,6 |
| 302 | FKBP11 | Homo sapiens FK506 binding protein 11, 19 kDa (FKBP11), mRNA. | 2,7 |
| 303 |  | Homo sapiens cDNA: FLJ23313 fis, clone HEP11919 | 2,5 |
| 304 | GPAA1 | Homo sapiens glycosylphosphatidylinositol anchor attachment protein 1 homolog (yeast) (GPAA1), mRNA. | 2,5 |
| 305 | FANCB | Homo sapiens Fanconi anemia, complementation group B (FANCB), transcript variant 1, mRNA. | 2,4 |
| 306 | SLC16A3 | Homo sapiens solute carrier family 16, member 3 (monocarboxylic acid transporter 4) (SLC16A3), transcript variant 2, mRNA. | 2,6 |
| 307 | HLA-C | Homo sapiens major histocompatibility complex, class I, C (HLA-C), mRNA. | 2,4 |
| 308 | CCDC80 | Homo sapiens coiled-coil domain containing 80 (CCDC80), transcript variant 1, mRNA. | 2,4 |
| 309 |  | Homo sapiens cDNA clone IMAGE:3079901 | 2,4 |
| 310 | ALDOA | Homo sapiens aldolase A, fructose-bisphosphate (ALDOA), transcript variant 2, mRNA. | 2,4 |
| 311 | KCNN4 | Homo sapiens potassium intermediate/small conductance calcium-activated channel, subfamily N, member 4 (KCNN4), mRNA. | 2,4 |
| 312 | BAPX1 | Homo sapiens bagpipe homeobox homolog 1 (Drosophila) (BAPX1), mRNA. | 2,5 |
| 313 | PLOD2 | Homo sapiens procollagen-lysine, 2-oxoglutarate 5-dioxygenase 2 (PLOD2), transcript variant 2, mRNA. | 2,5 |
| 314 | ODF3L2 | Homo sapiens outer dense fiber of sperm tails 3-like 2 (ODF3L2), mRNA. | 2,5 |
| 315 | CD24 | Homo sapiens CD24 molecule (CD24), mRNA. | 2,6 |
| 316 |  | Homo sapiens cDNA clone IMAGE:6186815, partial cds | 2,4 |
| 317 | CDKN1A | Homo sapiens cyclin-dependent kinase inhibitor 1A (p21, Cip1) (CDKN1A), transcript variant 1, mRNA. | 2,4 |
| 318 | COMTD1 | Homo sapiens catechol-O-methyltransferase domain containing 1 (COMTD1), mRNA. | 2,4 |
| 319 | HLA-B | Homo sapiens major histocompatibility complex, class I, B (HLA-B), mRNA. | 2,7 |
| 320 | MAD1L1 | Homo sapiens MAD1 mitotic arrest deficient-like 1 (yeast) (MAD1L1), transcript variant 2, mRNA. | 2,4 |
| 321 | TGFBRAP1 | Homo sapiens transforming growth factor, beta receptor associated protein 1 (TGFBRAP1), mRNA. | 2,4 |
| 322 | LOC729252 | PREDICTED: Homo sapiens similar to Keratin, type I cytoskeletal 14 (Cytokeratin-14) (CK-14) (Keratin-14) (K14) (LOC729252), mRNA. | 2,4 |
| 323 | NR4A2 | Homo sapiens nuclear receptor subfamily 4, group A, member 2 (NR4A2), transcript variant 1, mRNA. | 2,7 |
| 324 | RORA | Homo sapiens RAR-related orphan receptor A (RORA), transcript variant 3, mRNA. | 2,4 |
| 325 | MAP1B | Homo sapiens microtubule-associated protein 1B (MAP1B), mRNA. | 2,4 |
| 326 | CRYBB2 | Homo sapiens crystallin, beta B2 (CRYBB2), mRNA. | 2,5 |
| 327 | GMFG | Homo sapiens glia maturation factor, gamma (GMFG), mRNA. | 2,4 |
| 328 | ADAM15 | Homo sapiens ADAM metallopeptidase domain 15 (ADAM15), transcript variant 2, mRNA. | 2,4 |
| 329 | FAM38A | Homo sapiens family with sequence similarity 38, member A (FAM38A), mRNA. | 2,4 |
| 330 | MAPRE3 | Homo sapiens microtubule-associated protein, RP/EB family, member 3 (MAPRE3), mRNA. | 2,4 |
| 331 | ZMAT3 | Homo sapiens zinc finger, matrin type 3 (ZMAT3), transcript variant 2, mRNA. | 2,3 |
| 332 | RPN2 | Homo sapiens ribophorin II (RPN2), mRNA. | 2,5 |
| 333 | MMP2 | Homo sapiens matrix metallopeptidase 2 (gelatinase A, 72kDa gelatinase, 72kDa type IV collagenase) (MMP2), mRNA. | 2,5 |
| 334 | PELI2 | Homo sapiens pellino homolog 2 (Drosophila) (PELI2), mRNA. | 2,4 |
| 335 | C6orf85 | Homo sapiens chromosome 6 open reading frame 85 (C6orf85), mRNA. | 2,4 |
| 336 | ATL2 | Homo sapiens atlastin GTPase 2 (ATL2), mRNA. | 2,3 |
| 337 | PZP | Homo sapiens pregnancy-zone protein (PZP), mRNA. | 2,4 |
| 338 | APBB1IP | Homo sapiens amyloid beta (A4) precursor protein-binding, family B, member 1 interacting protein (APBB1IP), mRNA. | 2,4 |
| 339 | CLEC11A | Homo sapiens C-type lectin domain family 11, member A (CLEC11A), mRNA. | 2,5 |
| 340 | FHL2 | Homo sapiens four and a half LIM domains 2 (FHL2), transcript variant 2, mRNA. | 2,4 |
| 341 | CRYBA2 | Homo sapiens crystallin, beta A2 (CRYBA2), transcript variant 1, mRNA. | 2,4 |
| 342 | WNT5A | Homo sapiens wingless-type MMTV integration site family, member 5A (WNT5A), mRNA. | 2,4 |
| 343 |  | Homo sapiens cDNA FLJ38512 fis, clone HCHON2000503 | 2,4 |
| 344 | FAM171A1 | Homo sapiens family with sequence similarity 171, member A1 (FAM171A1), mRNA. | 2,4 |
| 345 | OCIAD2 | Homo sapiens OCIA domain containing 2 (OCIAD2), transcript variant 1, mRNA. | 2,3 |
| 346 | CERCAM | Homo sapiens cerebral endothelial cell adhesion molecule (CERCAM), mRNA. | 2,7 |
| 347 | MYC | Homo sapiens v-myc myelocytomatosis viral oncogene homolog (avian) (MYC), mRNA. | 2,4 |
| 348 | SLC2A1 | Homo sapiens solute carrier family 2 (facilitated glucose transporter), member 1 (SLC2A1), mRNA. | 2,4 |
| 349 | DHCR7 | Homo sapiens 7-dehydrocholesterol reductase (DHCR7), transcript variant 1, mRNA. | 2,5 |
| 350 | TRAP1 | Homo sapiens TNF receptor-associated protein 1 (TRAP1), mRNA. | 2,3 |
| 351 | SH3KBP1 | Homo sapiens SH3-domain kinase binding protein 1 (SH3KBP1), transcript variant 1, mRNA. | 2,3 |
| 352 | AVPI1 | Homo sapiens arginine vasopressin-induced 1 (AVPI1), mRNA. | 2,3 |
| 353 | KIF26B | Homo sapiens kinesin family member 26B (KIF26B), mRNA. | 2,3 |
| 354 | CXXC5 | Homo sapiens CXXC finger 5 (CXXC5), mRNA. | 2,3 |
| 355 | FAM129B | Homo sapiens family with sequence similarity 129, member B (FAM129B), transcript variant 2, mRNA. | 2,3 |
| 356 | SFRS14 | Homo sapiens splicing factor, arginine/serine-rich 14 (SFRS14), transcript variant 1, mRNA. | 2,3 |
| 357 | MFF | Homo sapiens mitochondrial fission factor (MFF), nuclear gene encoding mitochondrial protein, mRNA. | 2,5 |
| 358 | GPR56 | Homo sapiens G protein-coupled receptor 56 (GPR56), transcript variant 2, mRNA. | 2,7 |
| 359 | DDR2 | Homo sapiens discoidin domain receptor tyrosine kinase 2 (DDR2), transcript variant 2, mRNA. | 2,3 |
| 360 | FLJ39632 | PREDICTED: Homo sapiens misc_RNA (FLJ39632), miscRNA. | 2,4 |
| 361 | ZNF259 | Homo sapiens zinc finger protein 259 (ZNF259), mRNA. | 2,3 |
| 362 | NT5DC2 | Homo sapiens 5'-nucleotidase domain containing 2 (NT5DC2), mRNA. | 2,4 |
| 363 | KRT86 | Homo sapiens keratin 86 (KRT86), mRNA. | 2,3 |
| 364 |  | AGENCOURT_14535501 NIH_MGC_191 Homo sapiens cDNA clone IMAGE:30415823 5, mRNA sequence | 2,6 |
| 365 | LOC653381 | PREDICTED: Homo sapiens similar to Sorbitol dehydrogenase (L-iditol 2-dehydrogenase) (LOC653381), mRNA. | 2,4 |
| 366 | YTHDC1 | Homo sapiens YTH domain containing 1 (YTHDC1), transcript variant 1, mRNA. | 2,3 |
| 367 | JPH1 | Homo sapiens junctophilin 1 (JPH1), mRNA. | 2,3 |
| 368 | SCARF2 | Homo sapiens scavenger receptor class F, member 2 (SCARF2), transcript variant 1, mRNA. | 2,3 |
| 369 | DNAJC12 | Homo sapiens DnaJ (Hsp40) homolog, subfamily C, member 12 (DNAJC12), transcript variant 2, mRNA. | 2,3 |
| 370 | NME1 | Homo sapiens non-metastatic cells 1, protein (NM23A) expressed in (NME1), transcript variant 1, mRNA. | 2,3 |
| 371 | NCALD | Homo sapiens neurocalcin delta (NCALD), mRNA. | 2,3 |
| 372 | SLC2A12 | Homo sapiens solute carrier family 2 (facilitated glucose transporter), member 12 (SLC2A12), mRNA. | 2,4 |
| 373 | NFIC | Homo sapiens nuclear factor I/C (CCAAT-binding transcription factor) (NFIC), transcript variant 2, mRNA. | 2,3 |
| 374 | INSIG2 | Homo sapiens insulin induced gene 2 (INSIG2), mRNA. | 2,4 |
| 375 | BYSL | Homo sapiens bystin-like (BYSL), mRNA. | 2,3 |
| 376 | PTPRF | Homo sapiens protein tyrosine phosphatase, receptor type, F (PTPRF), transcript variant 2, mRNA. | 2,3 |
| 377 | NEK6 | Homo sapiens NIMA (never in mitosis gene a)-related kinase 6 (NEK6), mRNA. | 2,4 |
| 378 | C22orf13 | Homo sapiens chromosome 22 open reading frame 13 (C22orf13), mRNA. | 2,3 |
| 379 | PLOD2 | Homo sapiens procollagen-lysine, 2-oxoglutarate 5-dioxygenase 2 (PLOD2), transcript variant 2, mRNA. | 2,4 |
| 380 | ADORA2B | Homo sapiens adenosine A2b receptor (ADORA2B), mRNA. | 2,3 |
| 381 | ITFG3 | Homo sapiens integrin alpha FG-GAP repeat containing 3 (ITFG3), mRNA. | 2,2 |
| 382 | CSNK1D | Homo sapiens casein kinase 1, delta (CSNK1D), transcript variant 1, mRNA. | 2,2 |
| 383 | PITX2 | Homo sapiens paired-like homeodomain 2 (PITX2), transcript variant 3, mRNA. | 2,2 |
| 384 | TUBB | Homo sapiens tubulin, beta (TUBB), mRNA. | 2,3 |
| 385 | EIF4EBP1 | Homo sapiens eukaryotic translation initiation factor 4E binding protein 1 (EIF4EBP1), mRNA. | 2,2 |
| 386 | FAM57A | Homo sapiens family with sequence similarity 57, member A (FAM57A), mRNA. | 2,5 |
| 387 | FOXQ1 | Homo sapiens forkhead box Q1 (FOXQ1), mRNA. | 2,4 |
| 388 | PCSK7 | Homo sapiens proprotein convertase subtilisin/kexin type 7 (PCSK7), mRNA. | 2,3 |
