# Supplementary material for: Similar Properties of Chondrocytes from Osteoarthritis Joints and Mesenchymal Stem Cells from Healthy Donors for Tissue Engineering of Articular Cartilage
Source: PLoS One. 2013 May 9;8(5):e62994. doi: 10.1371/journal.pone.0062994 (PMC3650033; doi:10.1371/journal.pone.0062994)
Supplement: Table S3 — Gene Ontology comparison results on OA chondrocytes (A) and chondrogenic differentiated human BM-MSCs (B). (DOC) [file pone.0062994.s003.doc]

**Supplementary Table S3.** Gene Ontology comparison results on osteoarthritis chondrocytes (**A**) and chondrogenic differentiated human bone marrow-MSCs (**B**)

**A**

| GO category/term | Genes in selection | BF p-value |
| --- | --- | --- |
| Cellular component | | |
| Cell | 254 | 9.38E-06 |
| Extracellular region | 82 | 5.82E-16 |
| Molecular function | | |
| Binding | 238 | 3.26E-14 |
| Biological process | | |
| Biological regulation | 147 | 8.69E-09 |
| Developmental process | 76 | 1.74E-02 |
| Signaling | 80 | 1.19E-06 |
| Response to stimulus | 91 | 1.50E-04 |
| Biological adhesion | 33 | 5.02E-06 |
| Cellular component organization  or biogenesis | 47 | 7.19E-03 |

**B**

| GO category/term | Genes in selection | BF p-value |
| --- | --- | --- |
| Cellular component | | |
| Cell | 275 | 2.28E-18 |
| Extracellular region | 64 | 5.53E-08 |
| Molecular function | | |
| Molecular transducer activity | 52 | 6.77E-03 |
| Binding | 234 | 1.73E-16 |
| Catalytic activity | 99 | 4.95E-02 |
| Biological process | | |
| Biological regulation | 151 | 3.04E-12 |
| Developmental process | 107 | 1.49E-15 |
| Multicellular organismal process | 99 | 4.68E-15 |
| Cellular process | 119 | 3.24E-04 |
| Signaling | 79 | 2.70E-07 |
| Response to stimulus | 104 | 1.98E-10 |
| Biological adhesion | 26 | 8.99E-03 |
| Cellular component organization  or biogenesis | 46 | 4.69E-03 |
